# IGF2BP3 promotes the proliferation and cisplatin resistance of bladder cancer by enhancing the mRNA stability of CDK6 in an m6A dependent manner

**DOI:** 10.7150/ijbs.103522

**Published:** 2025-02-18

**Authors:** Qiang Song, Wei Wang, Hao Yu, Zijian Zhou, Juntao Zhuang, Jiancheng Lv, Linjing Jiang, Xiao Yang, Qiang Lu, Haiwei Yang

**Affiliations:** Department of Urology, The First Affiliated Hospital of Nanjing Medical University, Nanjing 210029, PR China.

**Keywords:** IGF2BP3, bladder cancer, CDK6, m6A, chemotherapy

## Abstract

Cisplatin-based chemotherapy is a primary treatment for bladder cancer, yet the development of chemoresistance poses a significant therapeutic challenge. Insulin-like growth factor II mRNA binding protein 3 (IGF2BP3) is an RNA-binding protein and a key m6A reader that regulates various cancers through m6A-dependent mechanisms. However, its role in chemotherapy resistance in bladder cancer remains unclear. Our in *vivo* and in *vitro* experiments identified IGF2BP3 as a key regulator of cisplatin resistance in bladder cancer. We demonstrated that IGF2BP3 enhances the stability of CDK6 mRNA in an m6A-dependent manner, leading to increased CDK6 expression. This, in turn, promoted tumor cell proliferation and resistance to cisplatin chemotherapy. Moreover, we showed that the CDK6 inhibitor palbociclib effectively suppresses the pro-growth and chemoresistant effects induced by IGF2BP3 overexpression. These results suggested that the IGF2BP3/m6A/CDK6 axis plays a pivotal role in bladder cancer progression and chemoresistance, and that targeting this pathway with CDK6 inhibitors such as palbociclib may offer a promising therapeutic strategy for overcoming cisplatin resistance in bladder cancer.

## Introduction

Bladder cancer ranks among the top 10 prevalent malignant tumors worldwide with an estimated annual incidence of around 573,000 new cases and a mortality rate of about 213,000 1. Projections from the World Health Organization suggest that these numbers are anticipated to double by 2040 2. Bladder cancer comprises several subtypes including urothelial carcinoma - which is considered as predominant form - along with squamous cell carcinoma, sarcoma, lymphoma, and adenocarcinoma 3, 4. Additionally, bladder cancer can be classified into two main categories: non-muscle invasive bladder cancer (NMIBC)and muscle-invasive bladder cancer (MIBC), with NMIBC accounting for roughly 75% of all cases 5. Clinically, the cisplatin (CDDP)-based gemcitabine and cisplatin (GC) regimens have emerged as the established standard treatment for MIBC 6, 7. However, challenges still exist in terms of achieving long-lasting responses and overcoming chemoresistance in chemotherapy for bladder cancer.

N6-methyladenosine (m6A) is one of the most prevalent modifications in eukaryotic cells and has been identified as a post-transcriptional regulator of mRNAs, microRNAs, and lncRNAs 8-12. The m6A modification is dynamic and reversible, exerting its biological effects primarily through proteins known as "writers", "erasers", and "readers" 12. Dysregulation of epigenetic regulation is a significant contributor to the pathogenesis of bladder cancer 13, 14. Recent research has revealed that m6A modifications, including METTL3, YTHDF1, and FTO, play a pivotal role in promoting bladder cancer development and drug resistance 15-18. Furthermore, our previous research also revealed that METTL3 positively regulates the pri-miR221/222 process via an m6A-dependent mechanism to promote the development of bladder cancer 15. Additionally, Yu *et al.* demonstrated that the m6A demethylase ALKBH5 can impede the proliferation of bladder cancer cells while enhancing their sensitivity to cisplatin 19. As a crucial component of m6A modification, "reader" proteins possess the ability to recognize and bind these modifications, thereby executing diverse biological functions 20. Among them, IGF2BPs represent newly identified members of the "reader" family, encompassing IGF2BP1, IGF2BP2, and IGF2BP3 21. Belonging to the RNA-binding protein (RBP) family, IGF2BPs play a pivotal role in determining mRNA transcript fate 22. Among them, IGF2BP3 is reported to promote the stability and translation of the mRNA by binding to the m6A site in the non-coding region 23, which indirectly leads to many carcinogenic processes 24-26. Furthermore, IGF2BP3 has been found to be up-regulated in several types of tumors recently, and involved in tumor cell proliferation, invasion, chemotherapy resistance and associated with poor prognosis for patients 27, 28. However, the specific mechanism and potential function of IGF2BP3 in cisplatin resistance of bladder cancer remain unclear.

Abnormal regulation of the cell cycle is closely associated with the occurrence, progression, and prognosis of various malignant tumors^29^. Among them, cyclin-dependent kinase 4/6 (CDK4/6) exhibits abnormal activation during tumorigenesis and tumor development^30^. CDK4/6 activation facilitates the transition from G1 to S phase and confers tumor cells with the capability to effectively suppress cellular senescence and apoptosis^31^. For instance, in melanoma cells, overexpression of CDK6 can form a complex with the transcription factor c-JUN, thereby enhancing VEGF-A transcription or regulating EZH2 independently of E2F to promote angiogenesis^32^. Although Sathe A *et al.* reported that CDK6 is involved in promoting the progression of bladder cancer^33^, its potential role in bladder cancer chemotherapy resistance and its correlation with m6A are still unclear.

In our study, we conducted a comprehensive investigation into the landscape of m6A modification in bladder cancer and identified a distinct expression pattern of IGF2BP3 in this context. Notably, IGF2BP3 exhibited high expression levels in bladder cancer patients, establishing it as an independent prognostic factor for bladder cancer. Moreover, an elevated expression level of IGF2BP3 was found to enhance proliferation and heighten resistance towards chemotherapy in *vivo* and in *vitro*, through modulating CDK6-mRNA stability in an m6A-dependent manner. Finally, our findings provided compelling evidence that the combined administration of CDK4/6-targeting inhibitors palbociclib and cisplatin effectively counteracted the oncogenic effects of IGF2BP3 and overcomes chemotherapy resistance in bladder cancer. These results suggested that IGF2BP3 maybe promise as a novel prognostic biomarker and therapeutic target for bladder cancer, while highlighting the potential of palbociclib combination therapy as an innovative treatment strategy.

## Materials and Methods

### Online databases and associated analyses

TCGA-Bladder cancer (TCGA-BLCA, https://cancergenome.nih.gov/) contains 408 bladder cancer cases and 19 normal control cases. GSE166716 (GEO, https://www.ncbi.nlm.nih.gov/geo/) contains 12 bladder cancer cases and 12 paired normal control cases.

### Clinical specimens

Bladder tumor and adjacent normal tissue samples, were obtained from patients diagnosed with bladder cancer who underwent surgical procedures at the First Affiliated Hospital of Nanjing Medical University from 2015 to 2020. The cutoff date for follow-up was May 2023, with all patients providing informed consent prior to the utilization of clinical data. The present study also obtained approval from the Ethics Committee of the First Affiliated Hospital of Nanjing Medical University.

### Cell culture

The bladder cancer cell lines (T24, J82, UMUC3, 5637, and 253J) and a human ureteric epithelial immortalized cell line (SVHUC-1 cells) were obtained from the Chinese Academy of Sciences (Shanghai, China). The culture system was DMEM (Gibco, Thermo Fisher Scientific, USA), supplemented with 10% fetal bovine serum (BioIndustries, Israel) and 1% penicillin/streptomycin (Gibco, Thermo Fisher Scientific, USA). All cell lines were maintained in a humidified incubator with 5% carbon dioxide.

### Tissue microarray (TMA) and immunohistochemistry (IHC)

The TMA was constructed using 152 formalin-fixed, paraffin-embedded bladder cancer tissues. Immunohistochemistry (IHC) was performed on the TMA to detect the expression levels of IGF2BP3 and CDK6 proteins. Prior to IHC, the TMA underwent xylene and 100% ethanol treatment, followed by a reduction in ethanol concentration. After antigen extraction, the TMA was subjected to IHC using anti-IGF2BP3 antibody (diluted at 1:500; Abcam Institute, USA) or CDK6 (diluted at 1:500; Abcam Institute, USA) for blocking and staining purposes. This was followed by incubation with secondary antibodies (diluted at 1:2000 and subsequent application of the standard avidin biotinylated peroxidase complex assay). The clinical characteristics of patients in our TMA cohort were provided in **Table S1**. The IGF2BP3 and CDK6 immunostaining score were calculated as the sum of the score for the proportion of positively stained tumor cells (PP) and the score for staining intensity (SI) given by two pathologists blinded to the clinical parameters. PP was scored into four categories: 0 (< 5%, negative), 1 (5-25%, sporadic), 2 (25-50%, focal), 3 (> 51%, diffuse) and SI was scored on a scale of 0 to 3 (0, negative staining; 1, weak staining; 2, moderate staining; 3, strong staining). The final staining score was calculated by multiplying SI and PP score, resulting in a score value ranging from 0 to 9. The positive level of IHC staining was scored by two urologists and patients with different scores were divided into low- (0-3) and high-staining (4-9) groups.

### Cell transfection

The lentiviral constructs for IGF2BP3 gene knockdown or overexpression were obtained from HANBIO (Hanheng Biological Technology Company, China). The METTL3 knockdown lentiviral construct was from OBIO (Obio Technology Corp, China). Bladder cancer cells were seeded at 50% concentration in 6-well dishes and infected with the IGF2BP3 overexpression lentivirus (referred to as oeIGF2BP3), negative control (referred to as NC), IGF2BP3/METTL3 knockdown lentivirus (referred to as shIGF2BP3-1, shIGF2BP3-2, shMETTL3), or scramble control (referred to as shNC) respectively. Stable transduction pools were generated by selection with puromycin (4 μg/ml) for a duration of 2 weeks.

CDK6 and METTL3 siRNA and NC plasmid were purchased from HANBIO (Hanheng Biological Technology Company, China). Transfection was performed using the Liposome 3000 kit (Invitrogen, USA) according to the manufacturer's instructions.

### RNA extraction and quantitative real-time PCR (qRT-PCR)

Total RNA was extracted from cells using Trizol Regent (Invitrogen, USA). HiScript II (Vazyme, China) was employed for cDNA synthesis. While mRNA was subjected to qRT-PCR analysis using the StepOne Plus real-time PCR system (Applied Biological Systems, USA) or 480 (Roche, USA). The primers utilized for qRT-PCR are provided in **Table S2**.

### Western blot

After trypsinization of the cells, proteins were extracted using radioimmunoprecipitation (RIPA) buffer (Beyotime Institute of Biotechnology, China) containing protease inhibitors (Thermo Fisher Scientific Technology, USA), and quantified using a bicinchoninic acid (BCA) protein assay kit (Beyotime Institute of Biotechnology, China). Total proteins were separated by SDS-PAGE on a 10% gel and transferred to a polyvinylidene fluoride (PVDF) membrane (Millipore Sigma, USA). Following blocking with 5% skim milk at room temperature for 2 hours, primary antibodies against IGF2BP3 (1:1000; Abcam Institute, United States), METTL3 (1:1000, Abcam, United States), or CDK6(1:1000; Abcam Institute, United States) were incubated overnight at 4°C. After washing three times with Tris-buffered saline containing Tween 20 (TBST), the membrane was incubated with second antibody (diluted at 1:2000 and subsequent application of the standard avidin biotinylated peroxidase complex assay) for an additional 1.5 hours at room temperature. Band signals were detected using a chemiluminescence system (Bio-Rad Laboratories Inc, USA) and analyzed using Image LAB software.

### Cell proliferation assay

The pretreated cells were enumerated and seeded into 96-well plates at a density of 2×10^3^ (T24) cells/well or 2×10^3^ (UMUC3) cells/well. Cell proliferation was assessed using the cell counting kit 8 assay (CCK8, Japan Molecular Technology Corporation, Japan) after incubation for 24, 48, 72, and 96 hours. Following the manufacturer's instructions, absorbance at a wavelength of 490 nm was measured using an enzyme standard instrument (Biotech, USA) after incubating at 37 °C for 1 hour.

### Colony-formation assay

After pretreatment, cell vaccination was performed on 6-well plates (800 cells per well) for colony forming experiments. The cells were then cultured for either 1 or 2 weeks. Subsequently, the cells were fixed with paraformaldehyde for 30 minutes, washed with PBS, and stained with a solution of 0.1% crystal violet. In the inhibitor experiments, palbociclib (American chemical) were added at concentrations of 1 μM.

### Cell cycle assay

A total of 1×10^6^ cells was collected, washed with phosphate buffered saline (PBS) and fixed with 75% ethanol at -20 °C for 24 hours. Subsequently, the cells were washed twice with PBS and stained with propidium iodide for 30 min using the cell test plus DNA reagent kit (BD Bioscience, USA). Flow cytometry analysis was performed on these cells using flow cytometer (Becton Dickinson, USA) and Cell Quest Modfit software for data interpretation.

### Apoptosis assay

After 24 or 48 hours of cisplatin treatment (Tokyo Chemical Industry, Japan), the cells were trypsinized and stained with Annexin V-isochlorophycocyanin (APC) and propidium iodide (Fcmacs, China) for 30 minutes at 4 °C. Subsequently, flow cytometry analysis was performed to evaluate the ratio of apoptosis cells (Becton Dickinson, USA).

### IC50 determination

Transfected cells were trypsinized and seeded at a density of 5000 cells per well in 96-well plates. Three replicates were established. Then the plates were incubated overnight in a humidified incubator with 5% carbon dioxide. Subsequently, transfected cells were treated with cisplatin (Tokyo Chemical Industry, Japan) at concentrations of 64, 32, 16, 8, 4, 2 or 1 μM for a duration of 24 hours. Cell viability was assessed using the CCK-8 method following the manufacturer's instructions. Semi-inhibitory concentration values were calculated based on a probabilistic regression model. All experiments were independently performed three times. Suppression rate of cisplatin was 1 - (OD value of *x* μM/OD value of 0 μM) ×100%.

### mRNA stability assay

UMUC3 and T24 cells transfected with control lentivirus, IGF2BP3 overexpression lentivirus, or IGF2BP3 knockdown lentivirus were treated with 2 mg/mL Act D for 0, 2, 4, 6, 8, or 10 hours. Total RNA was collected and subjected to qRT-PCR analysis. The transcript levels of CDK6 were normalized to β-actin control levels, and the relative half-life of CDK6 was calculated.

### Dual-luciferase reporter assay

The adenine residue embedded within the consensus sequence, located closest to the translation termination codon in the CDK6 5'-UTR was mutated (5'-AAACU-3' to 5'-AAUCU-3'). Cells were transfected with plasmids containing the 5' -UTR of wild or mutant fragments from CDK6 using Invitrogen Lipofectamine 3000 (ThermoFisher Scientific, USA), following the manufacturer's protocol. After transfection, firefly and Rinella luciferase activities were measured continuously using the dual luciferase reporter Assay System (Promega, Massachusetts, USA) at a time point of 48 hours. Finally, the ratio of luminescence between firefly and Rinella luciferase was calculated.

### RNA immunoprecipitation (RIP) assay

The RIP testing was conducted in accordance with the instructions of the magna RIP RNA-binding protein immunoprecipitation kit (Millipore Sigma, USA). Briefly, magnetic beads were pre-incubated with anti-IGF2BP3, METTL3 or IgG antibodies prior to their addition to cell lysates. Subsequently, the bound complexes underwent thorough washing, elution, purification, and analysis via qRT-PCR. The precipitation of RNA enrichment was normalized relative to the input control.

### MeRIP-qRT-PCR assay

For the m6A RNA binding assay, we isolated RNA from bladder cancer cells that were stably transfected with shMETTL3 and shNC. The isolated RNA was subjected to RNase I treatment (Sigma-Aldrich, USA) and sonicated for 10 seconds on an ice-water mixture. Immunoprecipitation was carried out using a specific anti-m6A antibody (1:1000; Abcam, USA), which had been previously validated by magnetic Life Technologies Dynabeads (Thermo Fisher Scientific, USA). This immunoprecipitation step was performed in RIPA buffer using the Magna RIP RNA-Binding Protein Immunoprecipitation Kit (Millipore Sigma, USA), and the resulting complex was incubated with DNA fragment-free RNA. Subsequently, proteinase K (20 mg/ml) at 42°C was used for a duration of 1.5 hours. Finally, we extracted the RNA using phenol/chloroform/isoamyl alcohol extraction method and performed qRT-PCR analysis with CDK6 primers to normalize the input.

### Tumor xenograft model

The T24 cells were stably transfected with shIGF2BP3, or negative controls. Subsequently, approximately 1×10^7^ cells were subcutaneously injected into BALB/C nude mice (18-22g, 5 weeks of age in each group). The mice in cisplatin treatment group were intraperitoneally injected with cisplatin (2.5 mg/kg body weight, twice a week) from day 7 after tumor inoculation. At the same time, the control mice were intraperitoneally injected with the same volume of normal saline. Tumor growth was monitored weekly by measuring the width (W) and length (L) using a caliper. And the tumor volume (V) was calculated using the formula V = (W2×L)/2. After 4 weeks of injection, euthanasia was performed on the mice followed by removal and weighing of the tumors. The tumors were then fixed and embedded for immunohistochemical analysis. All animal experiments were conducted in accordance with institutional ethical guidelines for animal research approved by the Animal Management Committee of Nanjing Medical University (IACUC-2101028).

### Statistical analysis

Statistical analyses were conducted using R (version 4.0.0) or GraphPad Prism (version 9.0.0). The comparison between the two groups was performed using a double-tailed Student's t-test. Categorical data were assessed using the chi-square test. Survival curves were generated employing the Kaplan-Meier method, while survival data were analyzed through univariate and multivariate Cox regression analysis. *P* < 0.05 was considered statistically significant.

## Results

### Bioinformatics analysis identified IGF2BP3 as a core m6A regulator of bladder cancer

**Figure 1:**
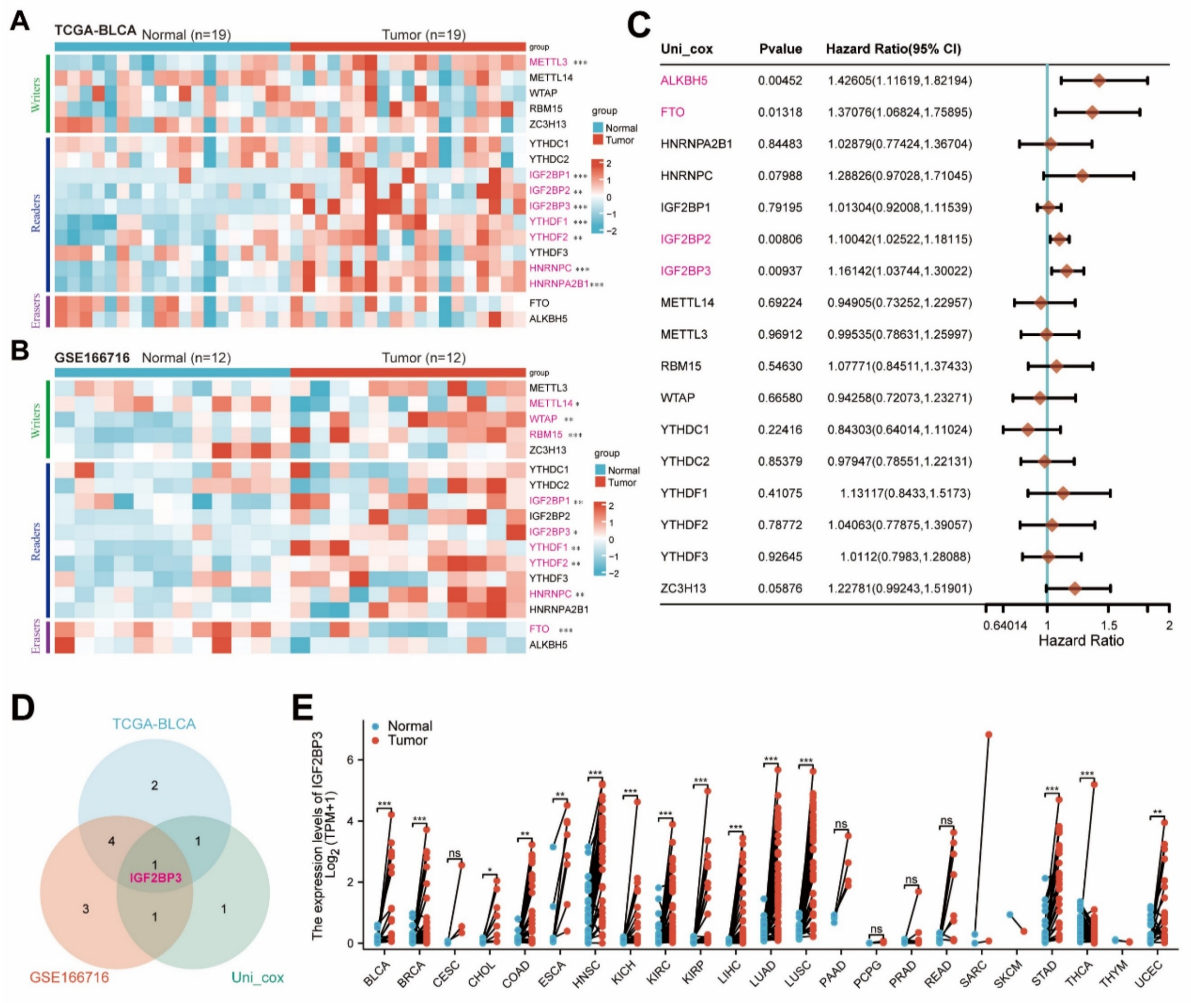
** Bioinformatics analysis identified IGF2BP3 as a core m6A regulator of bladder cancer.** (A-B) Consistently, IGF2BP1, IGF2BP3, YTHDF1, YTHDF2 and HNRNPC were significantly upregulated in bladder cancer tissues from TCGA-BLCA (A) and GSE166716 cohorts (B). (C) Univariate Cox regression analysis identified ALKBH5, FTO, IGF2BP2, and IGF2BP3 as potential risk factors for bladder cancer. (D) Venn diagram was generated to illustrate the overlapping genes, and confirmed that IGF2BP3 served as a core m6A regulator in bladder cancer. (E) TCGA pan-cancer dataset validated the high expression of IGF2BP3 across multiple cancers.

In order to comprehensively investigate the role of m6a-related genes in bladder cancer, we initially conducted bioinformatics analysis on publicly available TCGA-BLCA and GSE166716 datasets to determine the expression levels of these genes. The expressions of IGF2BP1, IGF2BP3, YTHDF1, YTHDF2, and HNRNPC were significantly up-regulated in bladder cancer **(Figure 1A-B)**. Subsequently, through univariate Cox regression analysis **(Figure 1C)**, we identified ALKBH5, FTO, IGF2BP2, and IGF2BP3 as potential risk factors for bladder cancer. To account for tissue-specific effects, a Venn diagram was generated to illustrate the overlapping genes. The differential genes obtained from TCGA-BLCA and GSE166716 datasets along with the identified risk factors for bladder cancer were included in this diagram for mapping purposes and showed IGF2BP3 was the only intersection gene **(Figure 1D)**. Finally, we validated the high expression of IGF2BP3 across multiple cancers using data from the TCGA pan-cancer dataset **(Figure 1E)**. Together, our findings confirmed that IGF2BP3 served as a core m6A regulator in bladder cancer**.**

### IGF2BP3 was highly expressed in bladder cancer and associated with poor prognosis of bladder cancer patients

**Figure 2:**
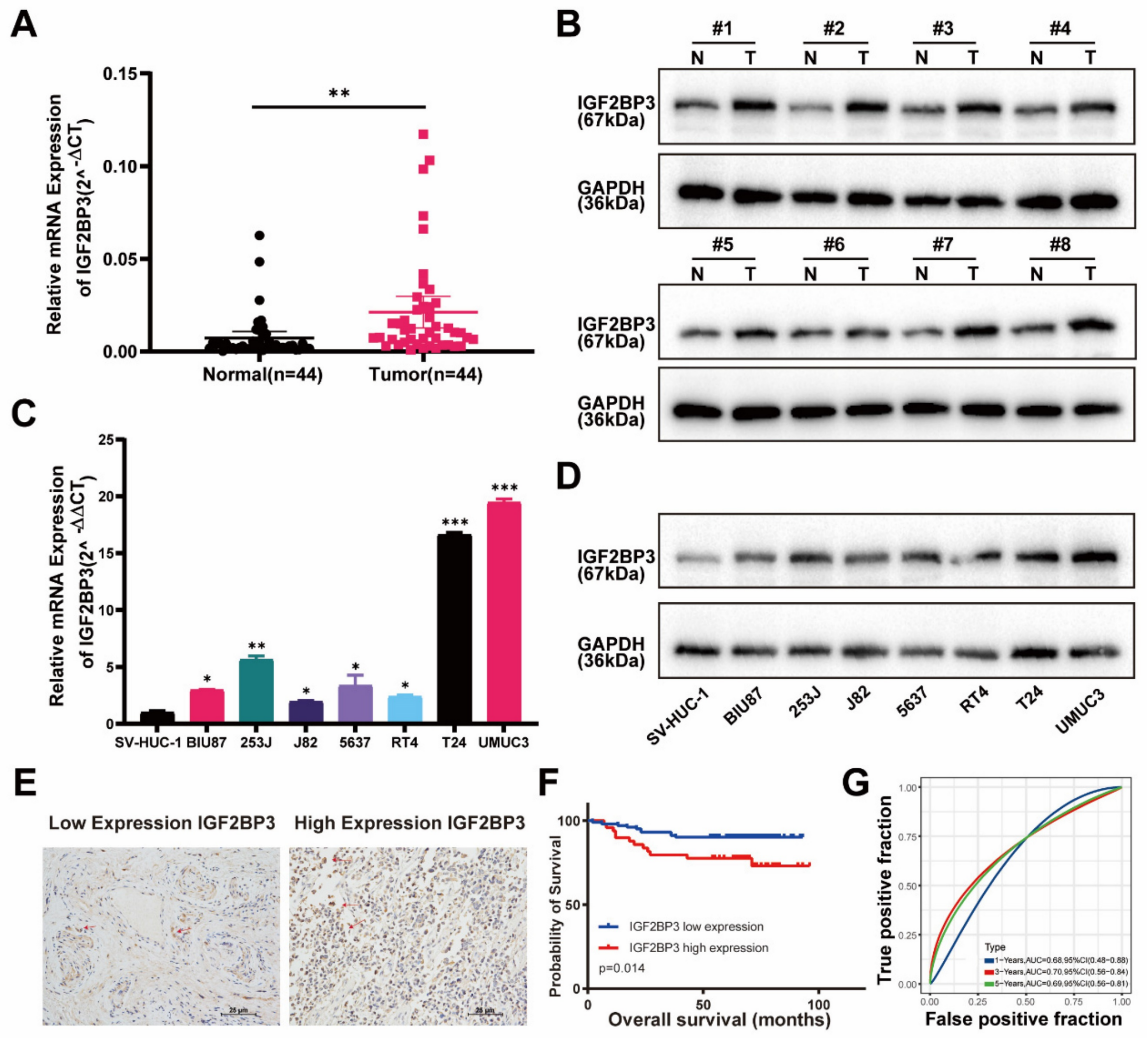
**IGF2BP3 was highly expressed in bladder cancer and associated with poor prognosis of bladder cancer patients.** (A) The mRNA expression of IGF2BP3 was significantly increased in bladder cancer tissues verified by qRT-PCR (**P< 0.01). (B) Protein expression of IGF2BP3 were significantly increased in bladder cancer tissues verified by western-blot (N: normal tissues; T: tumor tissues). (C-D) QRT-PCR (C) and western-blot (D) showed that compared to SV-HUC-1 (human ureteral epithelial immortalized cell line), IGF2BP3 was upregulated in bladder cancer cell lines (*P<0.05, **P<0.01, ***P<0.001). (E) Representative IHC pictures of low- and high-IGF2BP3 scores. (F-G) Bladder cancer patients with high expression of IGF2BP3 had poorer survival expectations based on the TMA cohort (P=0.014; F: Kaplan-Meier survive curve; G: receiver operating characteristic curve).

We then verified above findings in specimens from bladder cancer patients by RT-qPCR and western-blot. Indeed, the mRNA and protein expression of IGF2BP3 were significantly increased in tumor tissue compared with adjacent normal tissue of bladder cancer patients **(Figure 2A-B)**. IGF2BP3 expression was also upregulated in seven bladder cancer cell lines compared to SV-HUC-1 (human ureteral epithelial immortalized cell line) **(Figure 2C-D)**. We constructed TMA using clinical samples from 152 patients with bladder cancer. The relationships between the clinical and molecular characteristics with the IGF2BP3 expression level in patients with bladder cancer were listed in **Table 1.** According to the IHC score, 152 patients with bladder cancer were divided into 102 patients with low IGF2BP3 expression and 50 patients with high IGF2BP3 expression. Chi-square test analysis showed that the expression of IGF2BP3 in patients with bladder cancer was correlated with gender (P=0.025), but not with age, stage, grade, and tumor size. We showed representative IHC pictures of low- and high-IGF2BP3 scores **(Figure 2E)**. Further analysis based on the TMA cohort also found that bladder cancer patients with high expression of IGF2BP3 had poorer survival expectations (P=0.014) **(Figure 2F-G)**. Univariate and multivariate Cox regression models showed that high expression of IGF2BP3 was an independent risk factor for poor prognosis in patients with bladder cancer (HR>1, *P*<0.05) **Figure S1A-B)**. Taken together, IGF2BP3 was up-regulated in bladder cancer and predicted poor prognosis of bladder cancer patients.

### IGF2BP3 promoted the proliferation of bladder cancer cells

**Figure 3:**
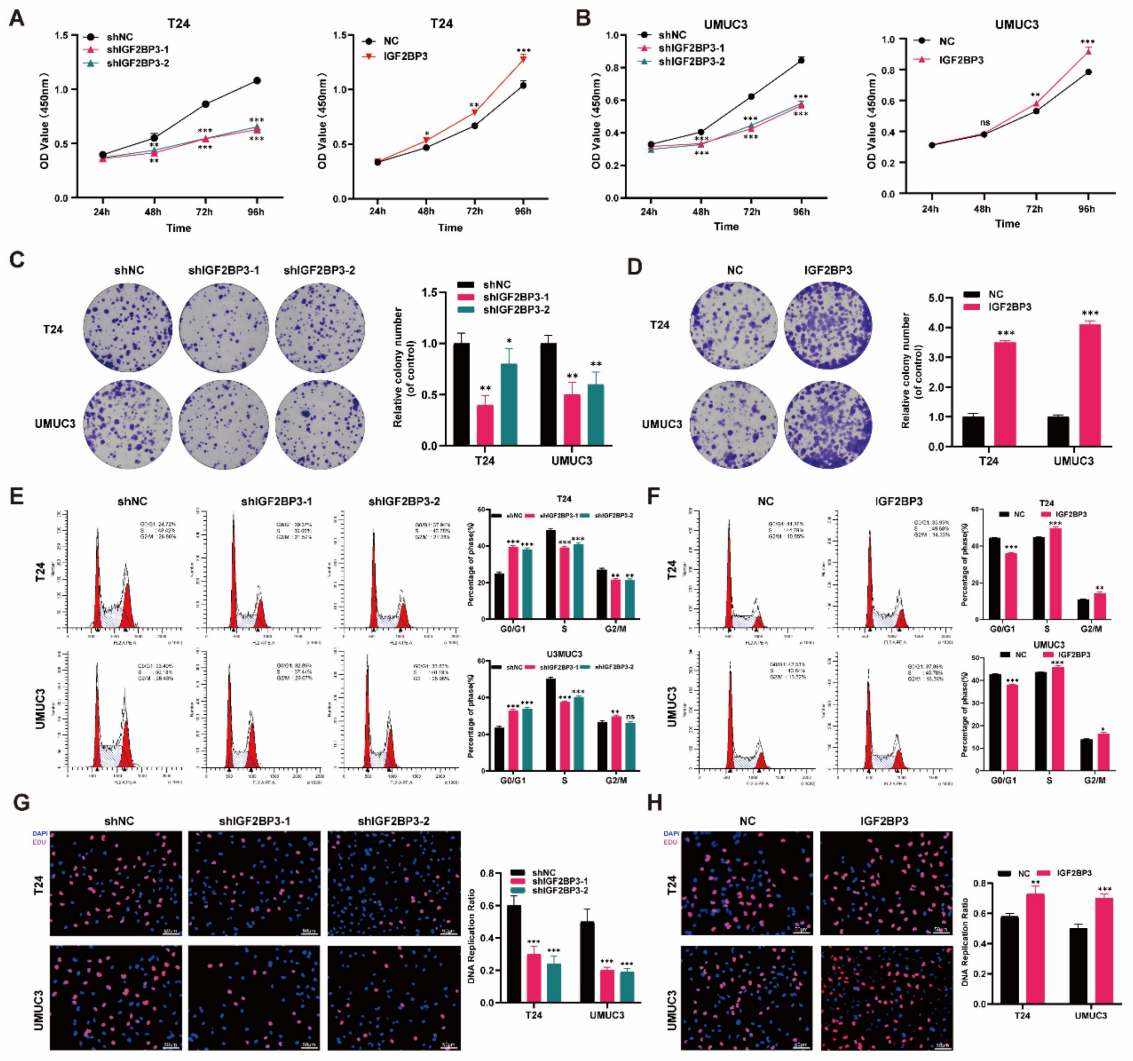
**IGF2BP3 promoted the proliferation of bladder cancer cells in* vitro.*** (A) Knockdown of IGF2BP3 significantly impeded the proliferative capacity of T24 and UMUC3 cells (**P<0.01, ***P<0.001). (B) Overexpression of IGF2BP3 augmented T24 and UMUC3 cell proliferative ability (*P<0.05, **P<0.01). (C) Knockdown of IGF2BP3 hindered colony formation (*P<0.05, **P<0.01). (D) Overexpression of IGF2BP3 led to increased colony formation rates(***P<0.001). (E) Flow cytometry analysis revealed that knockdown of IGF2BP3 elevated the proportion of G1 phase cells (**P<0.01, ***P<0.001). (F) Overexpression of IGF2BP3 reduced the proportion of G1 phase cells (*P<0.05, **P<0.01, ***P<0.001). (G) A significant inhibition in DNA replication capacity upon knockdown of IGF2BP3 in T24 and UMUC3 cell lines (***P<0.001). (H) DNA replication capacity upon overexpressing of IGF2BP3 in T24 and UMUC3 cell lines was upregulated (**P<0.01, ***P<0.001).

To further elucidate the role of IGF2BP3 in bladder cancer proliferation and chemotherapy sensitivity, we stably transfected T24 and UMUC3 bladder cancer cells with lentivirus knockout, lentivirus overexpression, or control lentivirus constructs. The transfection efficiency of IGF2BP3 was validated using qRT-PCR and western blot detection techniques **(Figure S2A-D)**. Subsequently, CCK-8 assay results demonstrated that knockdown of IGF2BP3 significantly impeded the proliferative capacity of T24 and UMUC3 cells. Conversely, overexpression of IGF2BP3 augmented their proliferative ability of bladder cancer cells** (Figure 3A-B)**. In colony formation experiments, knockdown of IGF2BP3 hindered colony formation **(Figure 3C)**, while its overexpression led to increased colony formation rates **(Figure 3D)**. Furthermore, flow cytometry analysis revealed that knockdown of IGF2BP3 elevated the proportion of G1 phase cells in bladder cancer cells; on the contrary, overexpression of IGF2BP3 exhibited an opposite trend** (Figure 3E-F)**. EDU assay was employed to assess DNA replication capacity in bladder cancer cells following either knockdown or overexpression of IGF2BP3. The findings indicated a significant inhibition in DNA replication capacity upon knockdown of IGF2BP3 in both T24 and UMUC33 cell lines. However, this effect was reversed by overexpressing IGF2BP3 **(Figure 3G-H)**. These results revealed the significant role of IGF2BP3 in promoting tumor cell proliferation.

### IGF2BP3 promoted chemotherapy resistance of bladder cancer cells to cisplatin in *vivo* and in *vitro*

**Figure 4:**
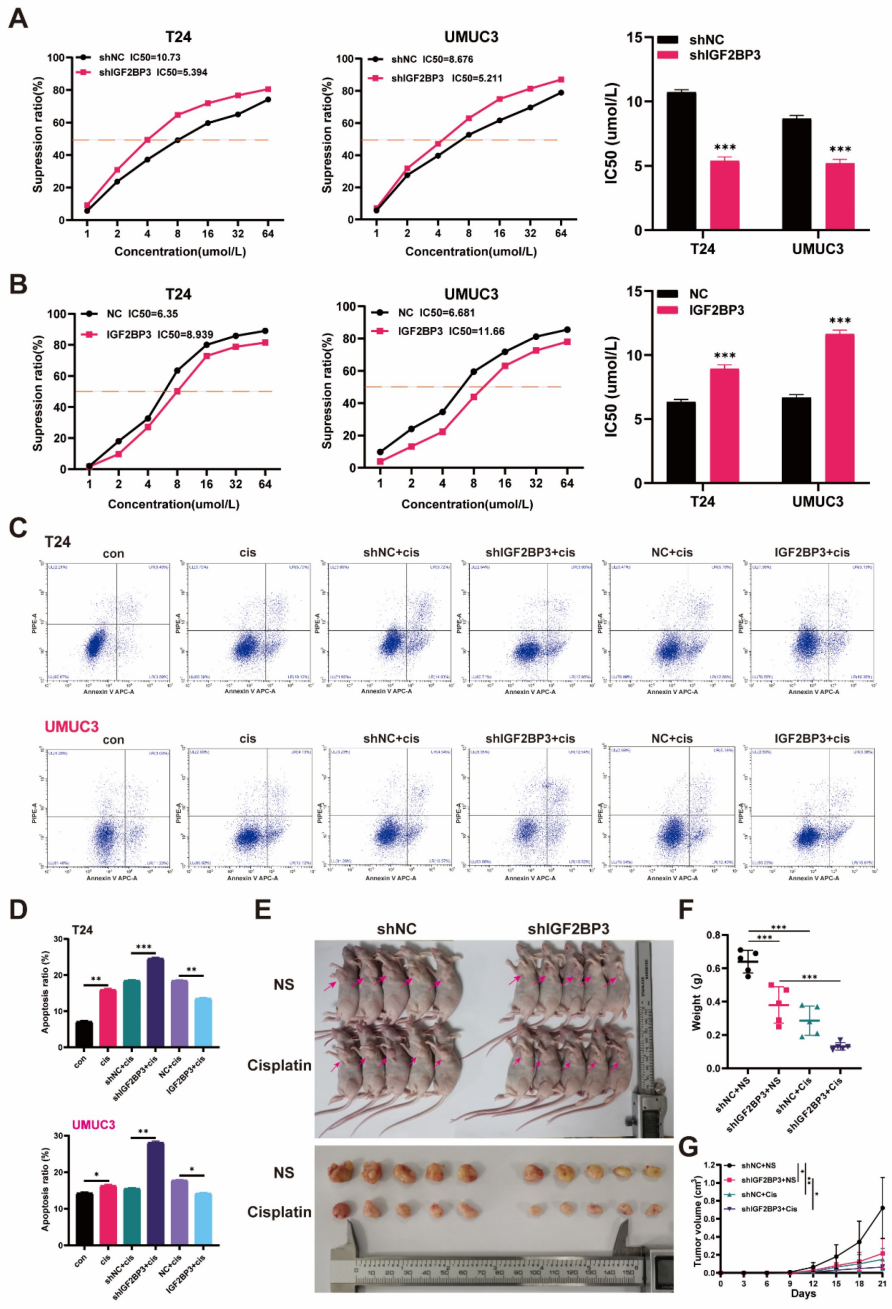
** IGF2BP3 promoted chemotherapy resistance of bladder cancer cells to cisplatin in *vivo* and in *vitro.*** (A) The cisplatin sensitivity of bladder cancer cells in the IGF2BP3 knockdown group was significantly increased after cisplatin treatment (***P<0.001). (B) Overexpression of IGF2BP3 significantly decreased the sensitivity of bladder cancer cells to cisplatin (***P<0.001). (C-D) Flow cytometry showed that after cisplatin treatment, the apoptosis rate of IGF2BP3 knockdown bladder cancer cells increased significantly. Conversely, the proportion of apoptosis of bladder cancer cells overexpressing IGF2BP3 was significantly reduced (*P<0.05, **P<0.01, ***P<0.001). (E) The IGF2BP3 knockdown subcutaneously transplanted tumor model (shIGF2BP3) or control cells (shNC) were subjected to intraperitoneal injection of cisplatin or normal saline on the 7th day after tumor inoculation. The red arrow indicates the location of the tumor. (F-G) The tumor weight (F) and volume (G) were significantly reduced in the IGF2BP3 knockdown group (shIGF2BP3) compared with the normal saline control group (shNC) (*P<0.05, **P<0.01, ***P<0.001).

To confirm the effect of IGF2BP3 on chemotherapy sensitivity of bladder cancer, CCK-8 and flow cytometry were used to detect the effect of IGF2BP3 on cisplatin sensitivity of bladder cancer cells. CCK-8 detection results showed that after cisplatin treatment, the sensitivity of bladder cancer cells in the IGF2BP3 knockdown group was significantly increased **(Figure 4A)**, while overexpression of IGF2BP3 significantly decreased the sensitivity of bladder cancer cells to cisplatin **(Figure 4B)**. Meanwhile, flow cytometry showed that after cisplatin treatment, the apoptosis rate of IGF2BP3 knockdown bladder cancer cells increased significantly. Conversely, the proportion of apoptosis of bladder cancer cells overexpressing IGF2BP3 was significantly reduced, further confirming that IGF2BP3 knockdown can increase the sensitivity of bladder cancer to cisplatin chemotherapy **(Figure 4C-D)**. Finally, the IGF2BP3 knockdown T24 cells or control group were injected under the skin of nude mice to establish an animal model to observe whether the expression of IGF2BP3 in animals affected the sensitivity of bladder cancer to cisplatin **(Figure 4E)**. The results showed that the tumor volume and weight were significantly reduced in the IGF2BP3 knockdown group (shIGF2BP3) compared with the normal saline control group (shNC). At the same time, after cisplatin administration, the tumor volume and weight of nude mice in IGF2BP3 knockdown group decreased compared with the normal saline control group **(Figure 4F-G)**. Taken together, these results suggested that IGF2BP3 knockdown could increase cisplatin chemotherapy sensitivity of bladder cancer in *vitro* and in *vivo*.

### CDK6 identified as the targeted genes of IGF2BP3 in bladder cancer

**Figure 5:**
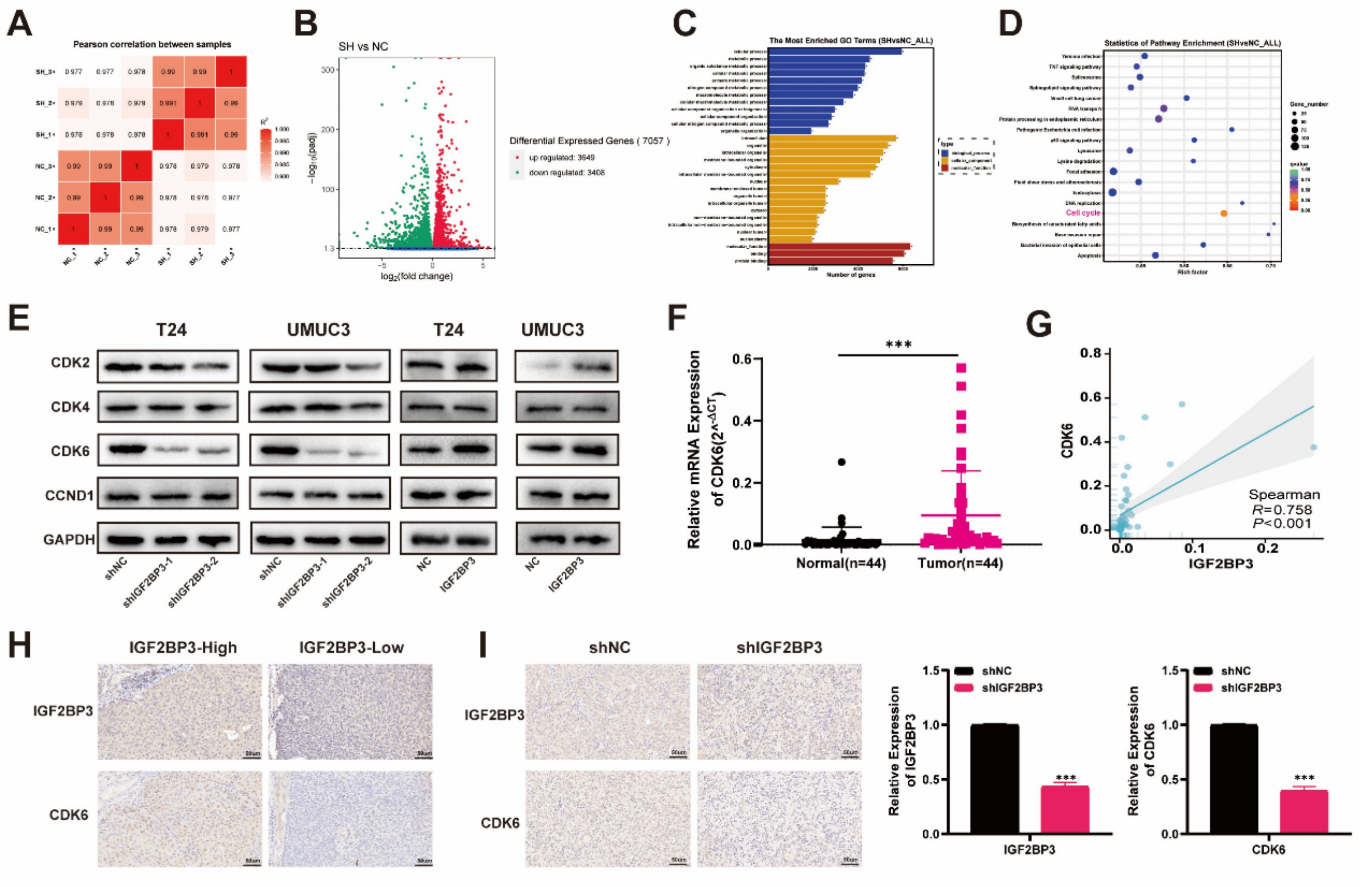
** CDK6 was identified as the targeted gene of IGF2BP3 in bladder cancer.** (A) RNA-seq analysis was conducted on T24 cells with IGF2BP3 knockdown and control groups. The heat map demonstrated reduced inter-group variation among samples. (B) The volcano plot revealed that IGF2BP3 knockdown led to significant alterations in 7,057 genes across the entire genome, including upregulation of 3,649 genes (red) and downregulation of 3,408 genes (green). (C) GO enrichment analysis. (D) KEGG pathway analysis. (E) Western blot analysis was performed to assess the expression of key cell cycle regulators in T24 and UMUC3 cells with knockdown or overexpression of IGF2BP3. (F) The mRNA levels of CDK6 exhibited significant upregulation in bladder cancer tissues (***P<0.001). (G) A positive association between CDK6 expression and IGF2BP3 (R=0.758; *P* < 0.001) was observed. (H) A significant positive correlation between the expression of CDK6 and IGF2BP3 was observed in the IHC analysis of TMA from bladder cancer patients. (I) A significantly positive correlation between the expression levels of CDK6 and IGF2BP3 in the IHC analysis of xenograft tumor model (***P<0.001).

To investigate the potential mechanism underlying the role of IGF2BP3 in bladder cancer proliferation and chemotherapy resistance, we initially conducted RNA-seq analysis on T24 cells with IGF2BP3 knockdown and control groups, each consisting of three biological replicates. The heat map demonstrated reduced inter-group variation among samples** (Figure 5A)**. The volcano plot revealed that IGF2BP3 knockdown led to significant alterations in 7,057 genes across the entire genome, including upregulation of 3,649 genes and downregulation of 3,408 genes **(Figure 5B)**. GO enrichment analysis indicated that these differentially expressed genes were enriched in cellular metabolism, immune response, cell membrane physiology, and protein binding functions **(Figure 5C)**. KEGG pathway analysis elucidated cell cycle pathway was enriched and associated with IGF2BP3 in bladder cancer **(Figure 5D)**. To validate the association between IGF2BP3 and key regulators within the cell cycle pathway, survival analysis and co-expression analyses were performed using TCGA-BLCA bladder cancer dataset. Results demonstrated a significant correlation between overexpression of CDK6 and poor survival prognosis for patients with bladder cancer (*P*=0.039), while other regulators within this pathway did not exhibit any impact on patient survival outcomes. Furthermore, co-expression analysis also confirmed a strong relationship between expression levels of CDK6 and IGF2BP3 (R = 0.428, *P* < 0.001) **(Figure S3**.

Subsequently, we performed western blot analysis to assess the expression of key cell cycle regulators in T24 and UMUC3 cells with knockdown or overexpression of IGF2BP3. Our results revealed a strong correlation between IGF2BP3 and CDK6** (Figure 5E)**. So, we postulated that CDK6 was the most plausible target for IGF2BP3. To validate this hypothesis, we quantified mRNA levels of both IGF2BP3 and CDK6 in 44 pairs of bladder cancer tissues and adjacent normal tissues. Notably, CDK6 exhibited significant upregulation in bladder cancer tissues **(Figure 5F)**. Furthermore, correlation analysis demonstrated a positive association between CDK6 expression and IGF2BP3 (R=0.758; *P* < 0.001) **(Figure 5G)**. The IHC analysis of TMA from bladder cancer revealed a significant positive correlation between the expression of CDK6 and IGF2BP3 **(Figure 5H; Table 1)**. Consistently, there was a significantly positive correlation between the expression levels of CDK6 and IGF2BP3 in the IHC analysis of xenograft tumor model **(Figure 5I)**. Collectively, these findings suggested that IGF2BP3 positively regulated the expression of CDK6 thereby influencing the cell cycle dynamics.

### IGF2BP3 increased the stability of CDK6 mRNA in an m6A-dependent manner

**Figure 6:**
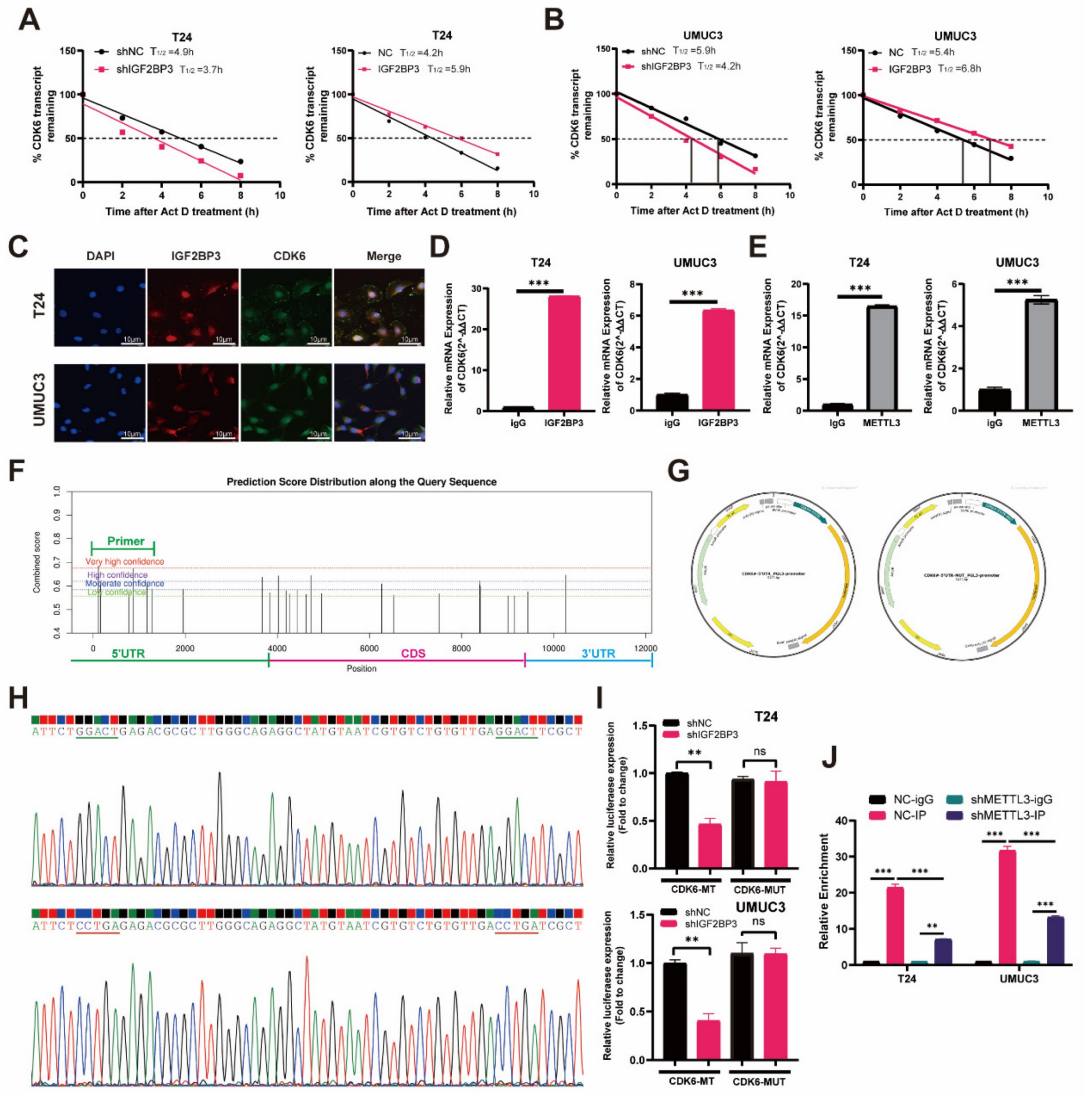
**IGF2BP3 increased the stability of CDK6 mRNA in an m6A-dependent manner.** (A) Depletion of IGF2BP3 resulted in a significant reduction in the half-life of CDK6 mRNA after Act D treatment. (B) Overexpression of IGF2BP3 extended the half-life of CDK6 mRNA after Act D treatment. (C) Immunofluorescence analysis revealed widespread distribution and high co-localization between IGF2BP3 and CDK6 in both the nucleus and cytoplasm. (D-E) RIP assay demonstrated that anti-IGF2BP3 antibodies significantly enriched CDK6 mRNA compared to IgG antibodies(***P<0.001). (F) The highest confidence was observed for binding at the 5'-UTR region of CDK6 in Starbase database (https://starbase.sysu.edu.cn). (G-H) We introduced mutations in the m6A modification site within the 5'-UTR of CDK6 and generated dual-luciferase reporter gene mutant plasmids along with wild-type and empty plasmid controls. (I) A decrease in IGF2BP3 binding to the 5'-UTR region of CDK6 upon transfection with wild-type plasmids following knockdown of IGF2BP3. However, knockdown of IGF2BP3 had no effect on its ability to bind to CDK6 after transfection with mutant plasmids (**P<0.01). (J) Me-RIP assay demonstrated that depletion of METTL3 led to reduced m6A levels associated with CDK6 in T24 cells and UMUC3 cells (**P<0.01).

We investigated whether IGF2BP3 regulated CDK6 expression by modulating the stability of CDK6 mRNA in bladder cancer cells. Indeed, depletion of IGF2BP3 resulted in a significant reduction in the half-life of CDK6 mRNA. Conversely, overexpression of IGF2BP3 extended the half-life of CDK6 mRNA **(Figure 6A-B)**. These findings suggested that IGF2BP3 can enhance the stability of CDK6 mRNA. Immunofluorescence analysis revealed widespread distribution and high co-localization between IGF2BP3 and CDK6 in both the nucleus and cytoplasm, consistent with the results obtained from immunohistochemistry **(Figure 6C)**. Subsequently, RIP assay demonstrated that anti-IGF2BP3 antibodies significantly enriched CDK6 mRNA compared to IgG antibodies **(Figure 6D)**. As a negative control, β-actin transcripts were not detected in either IGF2BP3 or IgG immune complexes. These results indicated that IGF2BP3 physically interacted with CDK6 transcripts. Therefore, we hypothesized that IGF2BP3 may bind to CDK6 mRNA in *vitro* and enhance its stability. Meanwhile, a positive correlation between METTL3 and CDK6 expression was also observed in METTL3 knockdown bladder cancer cell lines (T24 and UMUC3) **Figure S4A-B)**. RIP assay showed that METTL3 protein specifically bound to CDK6 mRNA **(Figure 6E)**. As CDK4 and CDK6 often exist in a complex form, it is of great value to explore the interaction between IGF2BP3 and CDK4. However, although IGF2BP3 and CDK4 showed a certain expression correlation in bladder cancer **(Figure S3**, further RIP experiments showed that the direct interaction between IGF2BP3 and CDK4 was not obvious **Figure S4C-D)**. Therefore, the mechanism of interaction between IGF2BP3 and CDK4 needs to be further explored.

Subsequently, to investigate the potential binding region of IGF2BP3 and CDK6, we utilized the Starbase database (https://starbase.sysu.edu.cn) for predicting IGF2BP3 binding sites on CDK6. Notably, our results demonstrated that the highest confidence was observed for binding at the 5'-UTR region of CDK6 **(Figure 6F)**. To further elucidate this interaction, we introduced mutations in the m6A modification site within the 5'-UTR of CDK6 and generated dual-luciferase reporter gene mutant plasmids along with wild-type and empty plasmid controls **(Figure 6G-H)**. Our findings revealed a decrease in IGF2BP3 binding to the m6A modification site within the 5'-UTR of CDK6 upon transfection with wild-type plasmids following knockdown of IGF2BP3 **(Figure 6I)**. However, knockdown of IGF2BP3 had no effect on its ability to bind to CDK6 after transfection with mutant plasmids **(Figure 6I)**. Additionally, employing m6A-RIP (me-RIP) assay demonstrated that depletion of METTL3 led to reduced m6A levels associated with CDK6 in T24 cells and UMUC3 cells **(Figure 6J)**. Collectively, these results suggested that IGF2BP3 enhanced the stability of CDK6 mRNA in an m6A-dependent mechanism.

### CDK6 interference decreased the cell proliferation and cisplatin chemotherapy resistance induced by IGF2BP3 in bladder cancer cells

To verify the role of CDK6 in bladder cancer, we transfected small interfering RNA (siCDK6) or control (SCR) plasmids of CDK6 into T24 and UMUC3 cells. The transfection efficiency of CDK6 interference was verified by qRT- PCR and western blot assays **(Figure S5A-B)**. CCK-8 assay results showed that knocking down CDK6 resulted in a significant decrease in cell growth rate **(Figure S5C)**. The colony-formation assays also produced consistent results **(Figure S5D)**. In addition, drug susceptibility assays had shown that knockdown of CDK6 can increase cisplatin chemotherapy sensitivity in bladder cancer cells **(Figure S5E)**.

**Figure 7:**
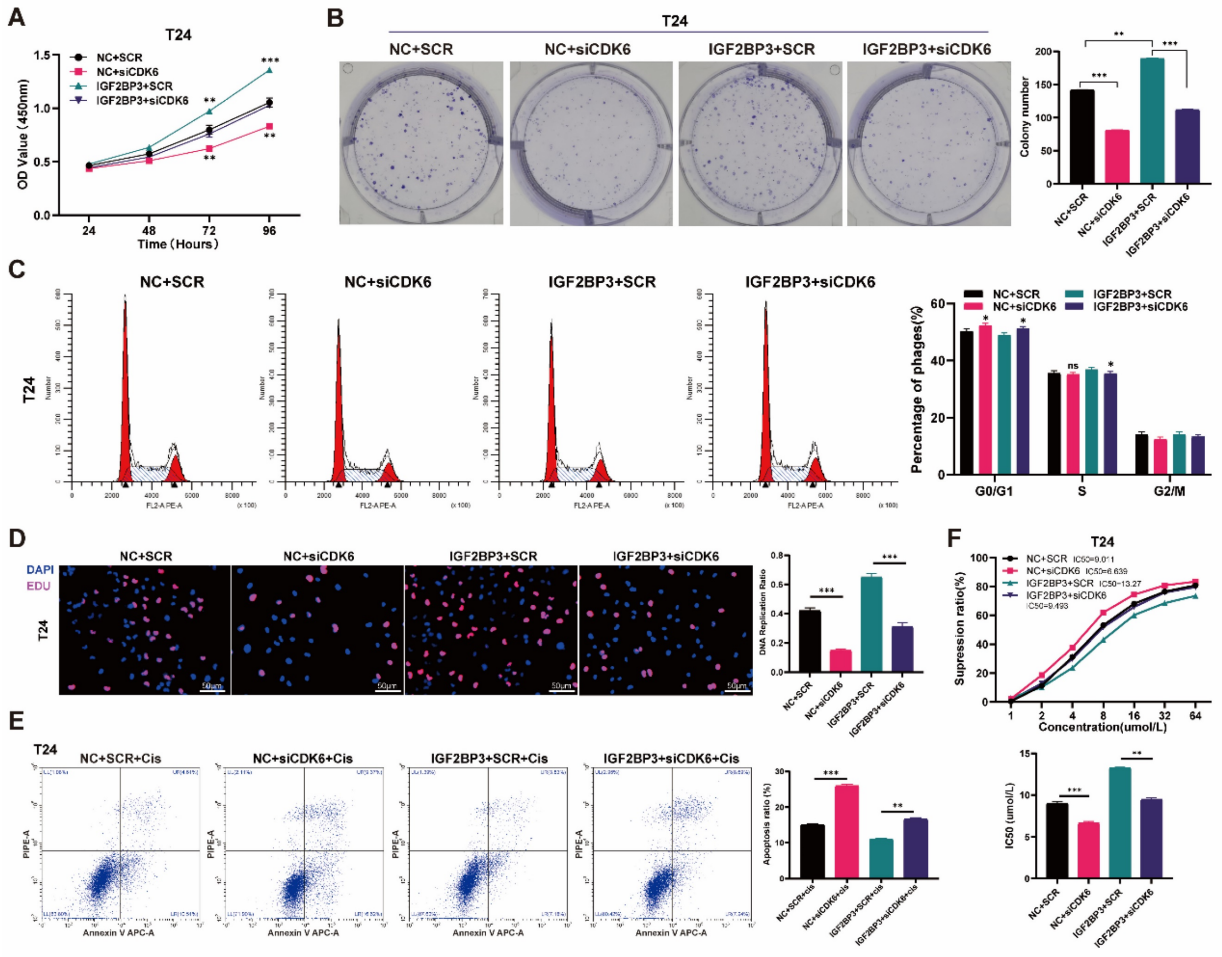
** CDK6 interference decreased the cell proliferation and increased the extent of apoptosis induced by IGF2BP3 in T24 cells.** (A) We transfected CDK6 small interfering RNA (siCDK6) or control (SCR) into over-expressed IGF2BP3 cells. CCK-8 assay results showed that knockdown of CDK6 reversed IGF2BP3 induced proliferation in T24 (**P<0.01, ***P<0.001). (B) Cloning experiments produced consistent results to CCK-8 assay (***P<0.001). (C) Knocking down CDK6 reversed G1 phase arrest induced by overexpression of IGF2BP3 (*P<0.05). (D) EDU assay demonstrated that knockdown of CDK6 gene expression reduced the DNA replication capacity of T24 induced by overexpression of IGF2BP3 (***P<0.001). (E) Knockdown of CDK6 could significantly increase the apoptosis rate of bladder cancer cells induced by IGF2BP3 (**P<0.01, ***P<0.001). (F) Knocking down CDK6 expression could significantly reduce the IC50 of cisplatin in T24 that were elevated due to over-expression of IGF2BP3 (**P<0.01, ***P<0.001).

To evaluate cell proliferation when CDK6 interacted with IGF2BP3 in bladder cancer, we transfected CDK6 small interfering RNA (siCDK6) or control (SCR) into over-expressed IGF2BP3 cells. CCK-8 assay results showed that knockdown of CDK6 reversed IGF2BP3 induced proliferation in bladder cancer cells **(Figure 7A and Figure S6A)**. Cloning experiments produced consistent results **(Figure 7B and Figure S6B)**. We also found that knocking down CDK6 reversed G1 phase arrest induced by overexpression of IGF2BP3 **(Figure 7C and Figure S6C)**. Similarly, EDU assay demonstrated that knockdown of CDK6 gene expression reversed the IGF2BP3 induced increase in DNA replication capacity of bladder cancer cells **(Figure 7D and Figure S6D)**.

When we explored the sensitivity of chemotherapy, flow cytometry demonstrated that knockdown of CDK6 could significantly increase the apoptosis rate of bladder cancer cells reduced by IGF2BP3 **(Figure 7E and Figure S6E)**. CCK-8 assay results also showed that knocking down CDK6 gene expression could significantly reduce the IC50 of cisplatin in bladder cancer cells that were elevated due to over-expression of IGF2BP3 **(Figure 7F and Figure S6F)**. Together, we confirmed that CDK6 interference decreased the cell proliferation and cisplatin resistance induced by IGF2BP3 in bladder cancer cells.

### The CDK4/6 inhibitor palbociclib reversed the proliferation of bladder cancer cells and cisplatin chemotherapy resistance induced by IGF2BP3

**Figure 8:**
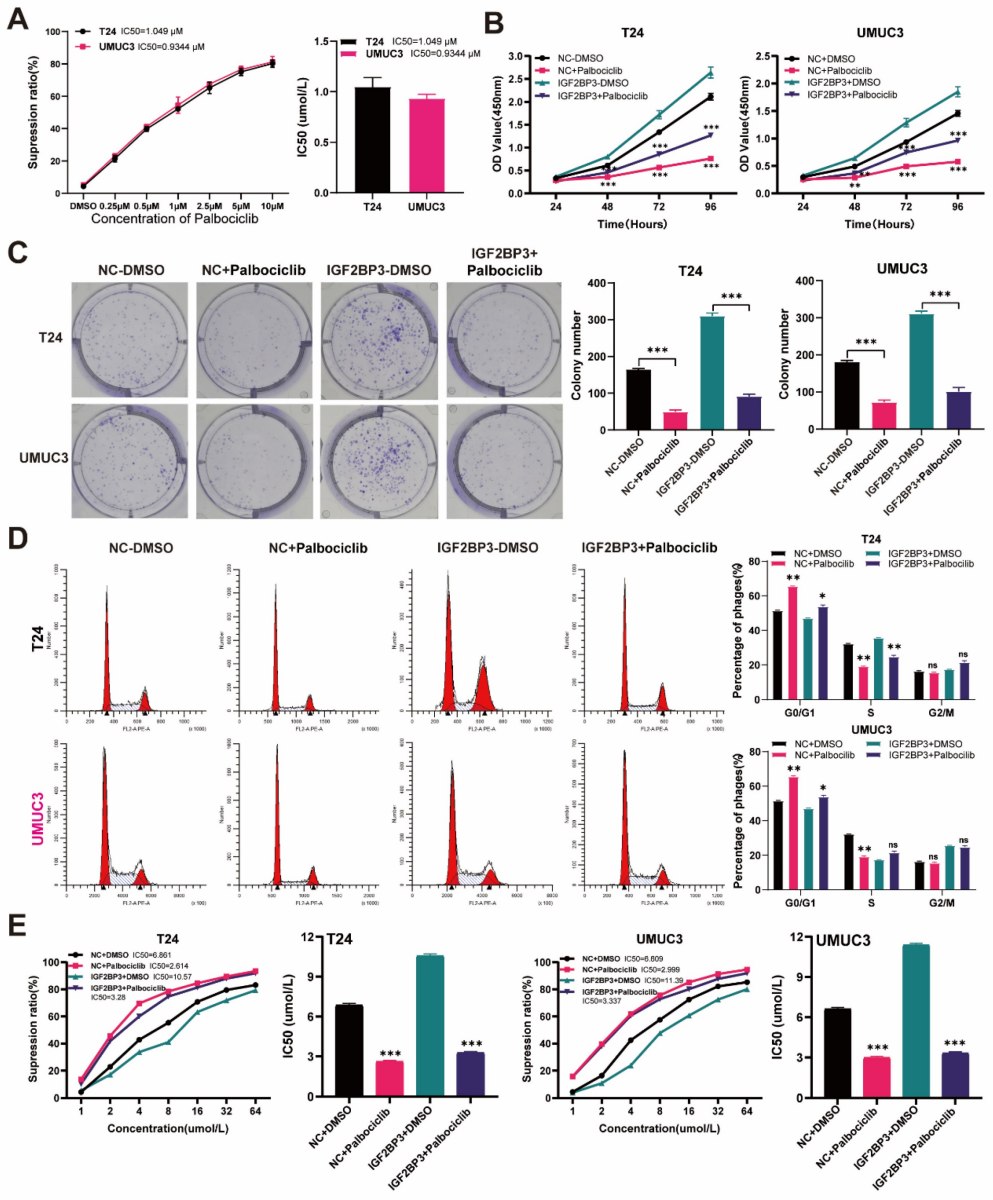
** Palbociclib reversed the proliferation of bladder cancer cells induced by IGF2BP3 over-expression and cisplatin chemotherapy resistance.** (A) Concentration-dependent experiments on palbociclib using T24 and UMUC3 cells. (B) The over-expressing IGF2BP3 or its control bladder cancer cells were treated with 1μM palbociclib. A significant inhibition in the proliferation of T24 and UMUC3 upon treatment with Palbociclib (**P<0.01, ***P<0.001). (C) Clonal formation experiments confirmed the results of the CCK8 proliferation assay (***P<0.001). (D) Palbociclib effectively reversed G1 phase arrest induced by IGF2BP3 overexpression (*P<0.05, **P<0.01). (E) The application of CDK6 inhibitor palbociclib can significantly mitigate the increase in cisplatin IC50 caused by IGF2BP3 overexpression (***P<0.001).

The full demonstration of palbociclib as a selective inhibitor of CDK4/6 has been achieved. According to a previous report (34, we performed concentration-dependent experiments on palbociclib using T24 and UMUC3 cells. The IC50 value of palbociclib in T24 cells was 1.049μM, while that in UMUC3 cells was 0.9344μm **(Figure 8A)**. Considering all these factors, we finally selected palbociclib at 1μM concentration for subsequent experimental verification. We supplemented the medium of over-expressing IGF2BP3 or its control bladder cancer cells with 1μM palbociclib. The results of the CCK8 assay revealed a significant inhibition in the proliferation of bladder cancer cells after treatment with palbociclib **(Figure 8B)**. This finding was further confirmed by clonal formation experiments **(Figure 8C)**. Moreover, we observed that palbociclib effectively reversed G1 phase arrest induced by IGF2BP3 **(Figure 8D)**. Additionally, cisplatin chemotherapy sensitivity experiments demonstrated that the application of CDK6 inhibitor palbociclib can significantly mitigate the increase in cisplatin IC50 caused by IGF2BP3 overexpression **(Figure 8E)**. To further verify this conclusion in *vivo*, T24 cells overexpressing IGF2BP3 or the control group were injected under the skin of nude mice to establish a xenograft tumor model, and to observe whether the expression of IGF2BP3 affects the sensitivity of bladder cancer to cisplatin and whether palbociclib enhances the sensitivity of bladder cancer to cisplatin in *vivo*. The results showed that compared with the control group (NC+NS), the tumor volume and weight of the IGF2BP3 overexpression group (IGF2BP3+NS) were significantly increased. Meanwhile, after cisplatin administration, the tumor volume and weight of nude mice in the IGF2BP3 overexpression group were not significantly reduced compared with the saline group, indicating the sensitivity of cisplatin chemotherapy inhibited by IGF2BP3. Importantly, simultaneous administration of palbociclib and cisplatin intraperitoneally significantly inhibited tumor growth in the IGF2BP3 overexpression group, indicating that palbociclib could improve sensitivity to cisplatin chemotherapy **Figure S7A-C)**. Concurrently, immunohistochemical (IHC) analysis was conducted to assess the expression profiles of IGF2BP3, CDK6, and Ki67 across different groups of xenograft tumor tissues. Notably, IGF2BP3 was highly expressed within the xenograft tumor tissues. Subsequent to the enforced overexpression of IGF2BP3, a marked upregulation in the expression levels of both CDK6 and Ki67 was observed. Intriguingly, upon administration of cisplatin, no significant attenuation in the expression of CDK6 and Ki67 was detected when compared to the negative control (NC+NS) group. However, the concomitant administration of the CDK6-specific inhibitor, palbociclib, elicited a profound suppression of CDK6 expression, concomitant with a significant diminution in Ki67 expression levels **(Figure S7D)**. In conclusion, palbociclib can increase the sensitivity of bladder cancer to cisplatin chemotherapy in *vitro* and in *vivo*. These results suggested that palbociclib also might have potential value in inhibiting bladder cancer cell proliferation and drug resistanceC.

## Discussion

**Figure 9:**
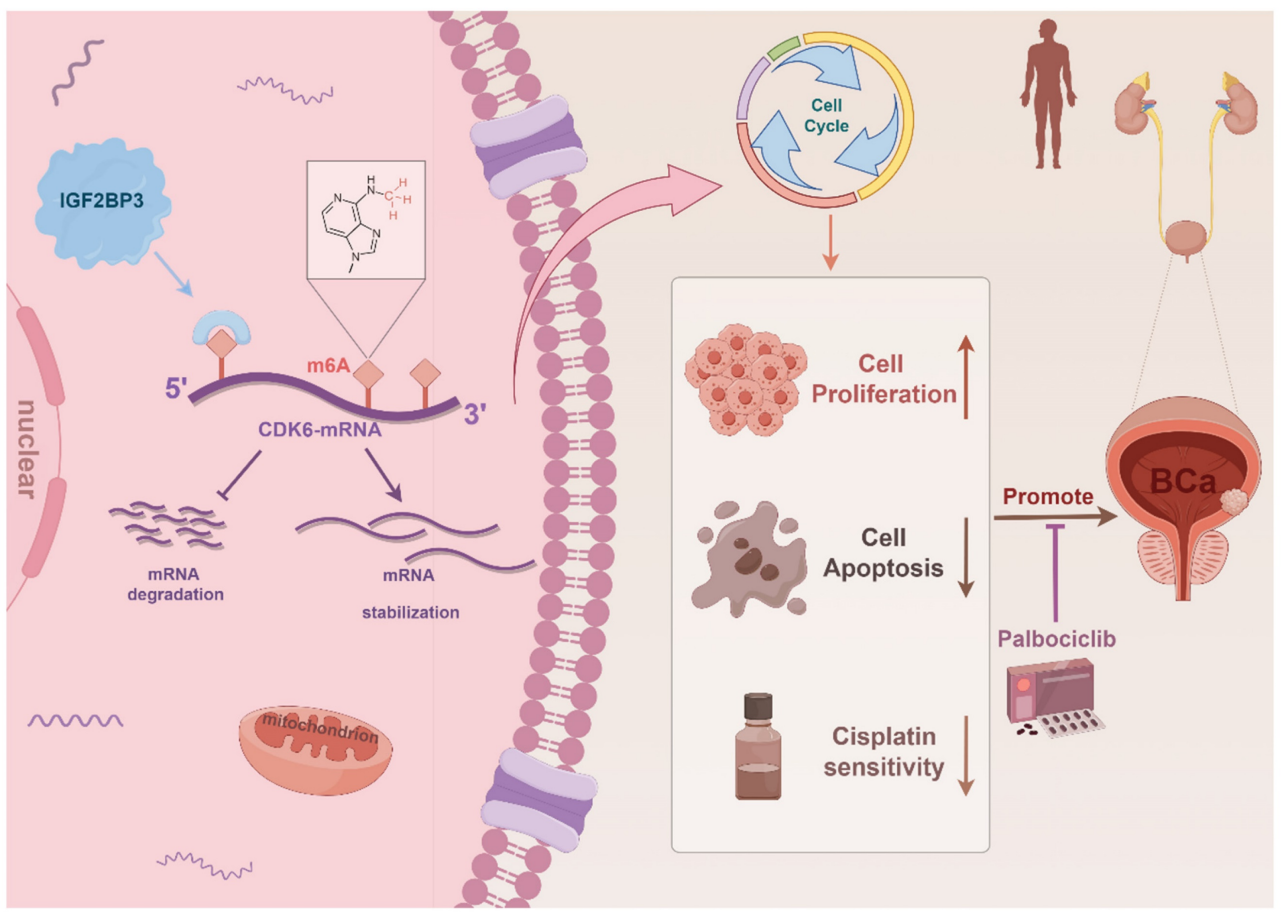
Schematic illustration of the mechanism by which IGF2BP3 facilitated bladder cancer tumorigenesis and conferred cisplatin resistance in an m6A manner.

As the most abundant RNA epigenetic modification, m6A modification dynamically and reversibly regulates RNA function (35. Dysregulation of enzymes involved in m6A modification is intricately associated with cancer progression encompassing tumorigenesis, metastasis, drug resistance, and angiogenesis 8, 36-38. In this study, we demonstrated that elevated expression of IGF2BP3 was associated with poor prognosis of bladder cancer patients. Moreover, IGF2BP3 promoted both the proliferation and cisplatin resistance of bladder cancer cells by binding to the 5'- UTR region of CDK6 mRNA and enhancing its stability in an m6A-dependent manner. Notably, CDK6 interference or treatment with the small molecule CDK6 inhibitor palbociclib effectively counteracted the pro-proliferative effect induced by IGF2BP3 while sensitizing bladder cancer cells to cisplatin. In conclusion, our study demonstrated that IGF2BP3 regulated the cell cycle and cisplatin sensitivity in bladder cancer by enhancing CDK6 mRNA stability in an m6A-dependent manner, which maybe providing a new treatment strategy for bladder cancer patients, especially those with cisplatin resistance **(Figure 9)**. Despite being a distinct m6A reader (unlike YTH domain family proteins which promote mRNA degradation but rather stabilize mRNA), there are no reports on its involvement in bladder cancer m6A regulation 21, 39. Thus, we aim to explore this aspect for the first time.

Accumulating evidence suggests that m6A exerts a dual role in cancer, encompassing the regulation of oncogenes or tumor suppressor genes to influence cancer progression, as well as the modulation of m6A levels and the expression and activity of m6A enzymes to impact its involvement in cancer 40-43. As an m6A reader, IGF2BP3 stands out as an oncoembryonic protein with strong affinity for RNA molecules 27, 44. While it is abundantly produced by tumors and fetal tissues, its expression is significantly down-regulated in adult tissues 22. Extensive research has demonstrated the involvement of IGF2BP3 in various cancers such as breast cancer, colon cancer, and non-small cell lung cancer 45-47. In this study, we initially identified aberrant regulation of IGF2BP3 in bladder cancer through high-throughput sequencing data analysis. Univariate Cox regression analysis revealed that IGF2BP3 was a significant predictor of poor prognosis in patients. Subsequently, our investigation on self-collected bladder cancer samples confirmed the overexpression of IGF2BP3 in tumor tissues. Furthermore, both univariate and multivariate Cox analyses demonstrated that IGF2BP3 served as an independent risk factor for unfavorable prognosis among bladder cancer patients, which is consist with Huang *et al.* previously reported 48. Survival analysis further substantiated that elevated expression levels of IGF2BP3 were associated with reduced patient survival rates. Additionally, immunohistochemical analysis indicated a correlation between increased IGF2BP3 expression and chemotherapy drug resistance in bladder cancer patients, suggesting its potential involvement in mediating resistance to chemotherapy. These findings initially validate the pivotal role of IGF2BP3 in bladder cancer development and chemoresistance. These findings suggest a unique role for IGF2BP3 in bladder cancer development.

RNA binding proteins play a crucial role in the regulation of RNA transcription, thereby influencing cancer development and treatment response 49-51. IGF2BP3, as an important RBP, has been reported to interact with ELAVL1 to recognize cell cycle and cell proliferation related genes, and lead to prolonged half-life of mRNA molecules and increased expression of target genes, thereby promoting colorectal cancer cell proliferation 52. Meanwhile, IGF2BP3 enhanced the stability of oncogene HMGB1 by binding to its mRNA and promoted the expression of HMGB1 in bladder cancer 53. In addition, as an important "reader" of m6A, IGF2BP3 was shown to target thousands of mRNA transcripts by recognizing a consensus sequence of RRACH (R corresponds to G or A; H corresponds to A, C or U), promoting the stability and translation of its target mRNAs 23. Zhang *et al.* reported that IGF2BP3 promotes the stability of RCC2 mRNA in an m6A-dependent manner by reading the m6A modification sites, thus promoting the progression of acute myeloid leukemia 28. However, the involvement of IGF2BP3 in m6A manner has not been reported in bladder cancer. In our study, we discovered that IGF2BP3 significantly governs bladder cancer cell proliferation both in *vivo* and in *vitro*, which is consistent with the findings of Huang and Lv *et al*.^53^, ^54^. In *vitro* experiments further corroborated the promotion of bladder cancer cell proliferation by IGF2BP3, as well as its contribution to cisplatin resistance. We performed RNA-seq sequencing analysis and identified cell cycle pathway which enriched and associated with IGF2BP3 in bladder cancer. Then, we performed WB experiments on bladder cancer cell lines with knockdown or overexpression of IGF2BP3 to investigate the expression changes of multiple cyclin proteins CDK2, CDK4, CDK6, and CCND1. Combined with the survival analysis of cell cyclin-related genes and their correlation analysis with IGF2BP3 expression in the TCGA-BLCA cohort and our clinical cohort, we found that CDK6 expression was positively correlated with IGF2BP3 expression in bladder cancer tissues and cell lines, and predicted poor prognosis in bladder cancer patients. CDK6, a crucial regulator of the cell cycle, forms a complex with CDK4 and binds to cyclin D to create the Cyclin D-CDK4/6 complex, which phosphorylates the Rb gene 55, 56. Subsequently, transcription factors are released to facilitate entry into the proliferative cycle 57. In certain instances, overexpression of CDK6 leads to excessive cell proliferation and tumor formation 58. Palanichamy* et al.* reported that IGF2BP3 enhances hematopoietic progenitor cell proliferation by binding and upregulating CDK6 expression 59. In our study, we demonstrated that IGF2BP3 and CDK6 colocalized in the cell membrane and nucleus. Meanwhile, IGF2BP3 regulates CDK6 mRNA stability by directly binding 5'-UTR regions of CDK6 mRNA. In our previous study, we have demonstrated that METTL3, as a key m6A modified methyltransferase, can positively regulate the methylation level of target genes in bladder cancer 15. Subsequent RIP and me-RIP experiments confirmed the specific recognition and binding of IGF2BP3 to the m6A modification site in the 5'-UTR of CDK6 mRNA in m6A-depend manner. This interaction enhances the stability of CDK6 mRNA, thereby regulating cell cycle progression and promoting bladder cancer cell proliferation.

Cisplatin, a chemotherapeutic drug used in the treatment of bladder cancer, is classified as a cell cycle non-specific agent 60, 61. Its mechanism of action involves forming cisplatin-DNA adducts that impair DNA synthesis and mitosis, leading to inhibition of DNA replication and transcription and ultimately inducing apoptosis in cancer cells 61-63. As an important part of the cell cycle, DNA replication occurs in S phase. G1-S phase arrest often leads to inhibition of DNA replication 64. Cisplatin-induced cell cycle arrest has been reported in multiple cancers, including bladder cancer 65, 66. Our findings demonstrated that IGF2BP3 enhances resistance to cisplatin in bladder cancer cells by interacting with CDK6, thereby impeding the cytotoxic effects of cisplatin. The m6A binding and stabilization of CDK6 by IGF2BP3 represents a potential molecular mechanism underlying cisplatin resistance in bladder cancer cells. In *vivo* experiments conducted on nude mouse models further confirmed the role of IGF2BP3 in promoting tumor growth and conferring resistance to cisplatin.

Since the 1980s, an enhanced comprehension of cancer biology and oncogenes has greatly facilitated the development of targeted therapies for cancer 67, 68. However, progress in targeted therapy for bladder cancer remains suboptimal. Therefore, there exists a critical need to explore novel therapeutic targets and develop innovative targeted drugs to enhance treatment outcomes for bladder cancer. Cyclin Dependent Kinase (CDK) 4/6 inhibitor, Palbociclib, developed by Pfizer, demonstrates its efficacy in the treatment of various tumors, including breast cancer. It exerts a specific cell cycle arrest in the G1 phase to impede tumor progression. Currently, it has obtained FDA approval for the treatment of ER+/HER2- postmenopausal advanced breast cancer 69. Our study revealed that palbociclib possesses the ability to overcome cisplatin resistance and suppress proliferation of bladder cancer cells characterized by elevated levels of IGF2BP3 protein expression. These findings underscore the potential clinical significance of targeting IGF2BP3 expression as a therapeutic strategy for bladder cancer.

In conclusion, our study underscores the pivotal role of IGF2BP3 in driving cisplatin resistance in bladder cancer, primarily by stabilizing CDK6 mRNA in an m6A-dependent manner. These findings not only establish IGF2BP3 as a critical mediator of chemotherapy resistance but also highlight its potential as a novel therapeutic target to overcome drug resistance in bladder cancer. Clinically, IGF2BP3 expression levels may serve as a valuable biomarker for predicting cisplatin resistance in bladder cancer patients, thereby facilitating the implementation of personalized treatment strategies. Moreover, targeting IGF2BP3 either through small molecule inhibitors or monoclonal antibodies represents a promising approach to resensitize bladder cancer cells to cisplatin, potentially improving treatment outcomes. When combined with CDK4/6 inhibitors, such as palbociclib, this strategy could yield synergistic effects, further enhancing therapeutic efficacy. Future research, clinical trials are essential to assess the feasibility and clinical applicability of IGF2BP3-targeted therapies in bladder cancer treatment.

## Supplementary Material

Supplementary figures.

Supplementary table 1.

Supplementary table 2.

## Figures and Tables

**Table 1:** Correlations between the expression of IGF2BP3 and clinicopathological features in bladder cancer patients.

Characteristics	Cases	IGF2BP3		*P* value
		Low	High	
All cases	152	102	50	
Age(years)				0.861
<65	59	39	20	
≥65	93	63	30	
Gender				0.025*
Male	116	73	43	
Female	36	29	7	
TNM stage				0.303
pTa-pT1	85	54	31	
pT2-pT4	67	48	19	
Histological grade				0.863
Low	69	47	22	
High	83	55	28	
Tumor size(cm)				0.228
<3	84	60	24	
≥3	68	42	26	
CDK6				
Low	76	71	5	<0.001***
High	76	31	45	

Statistically significant,* P* < 0.05

## Abbreviations

m6A: N6-methyladenosine
IGF2BP3: insulin-like growth factor II mRNA binding protein 3
MIBC: muscle-invasive bladder cancer
NMIBC: non-muscle invasive bladder cancer
TMA: tissue microarray
IHC: immunohistochemistry
RIP: RNA immunoprecipitation
CDK: cyclin dependent kinase
