# Supplementary material for: IGF2BP3 promotes the proliferation and cisplatin resistance of bladder cancer by enhancing the mRNA stability of CDK6 in an m6A dependent manner
Source: Int J Biol Sci. 2025 Feb 18;21(5):2048–66. doi: 10.7150/ijbs.103522 (PMC11900814; doi:10.7150/ijbs.103522)
Supplement: Supplementary file 1 — Supplementary figures. [file ijbsv21p2048s1.pdf]

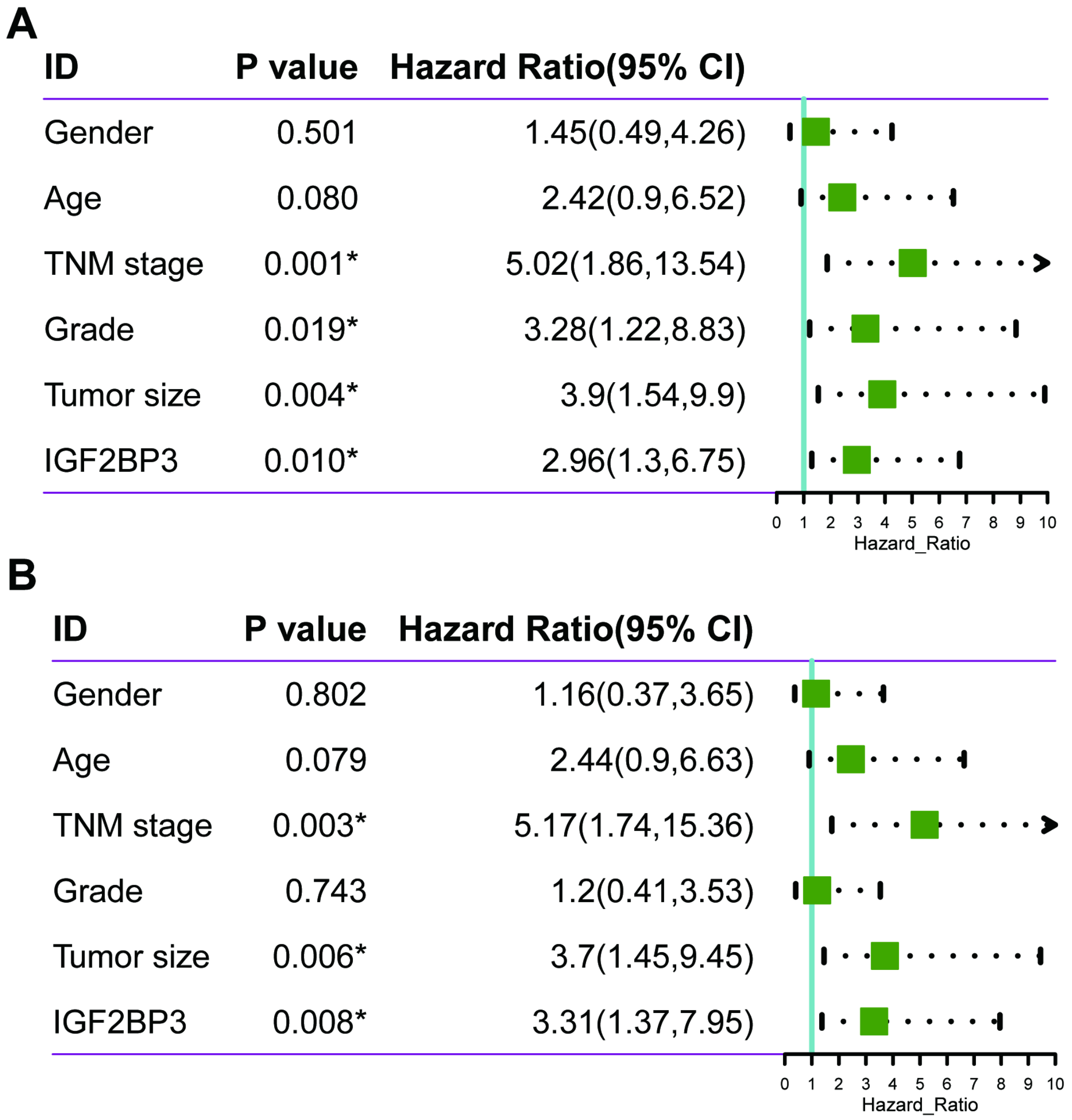

FigureS1: Bioinformatics analysis identified IGF2BP3 as an independent risk factor for poor prognosis of bladder cancer patients.

(A-B) Univariate (A) and multivariate (B) Cox regression models showed that high expression of IGF2BP3 was an independent risk factor for poor prognosis in patients with bladder cancer (HR>1, *P*<0.05).

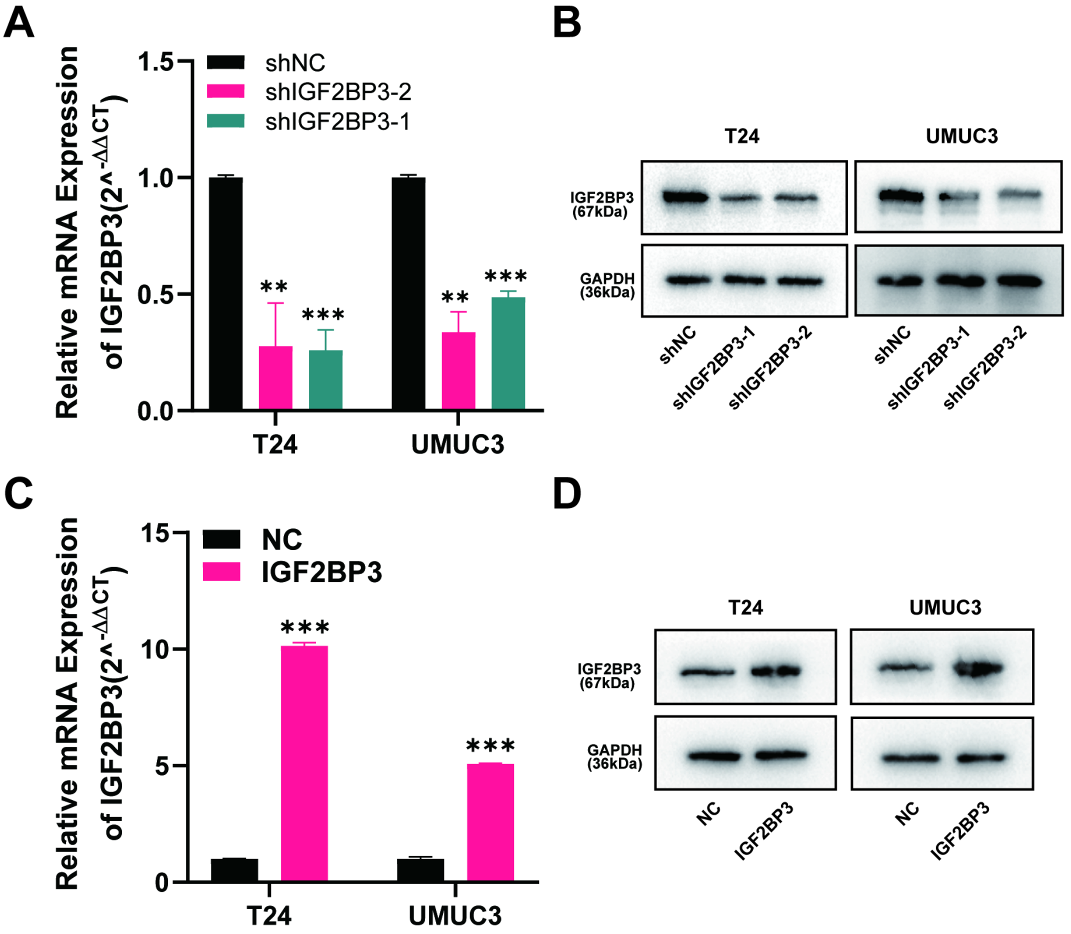

FigureS2: QRT-PCR and western-blot validated the transfection efficiency of IGF2BP3.

(A-B) High efficiency was achieved by interfering with IGF2BP3(***P*<0.01, ****P*< 0.001). (C-D) High efficiency was achieved by overexpressing IGF2BP3. (****P*< 0.001)

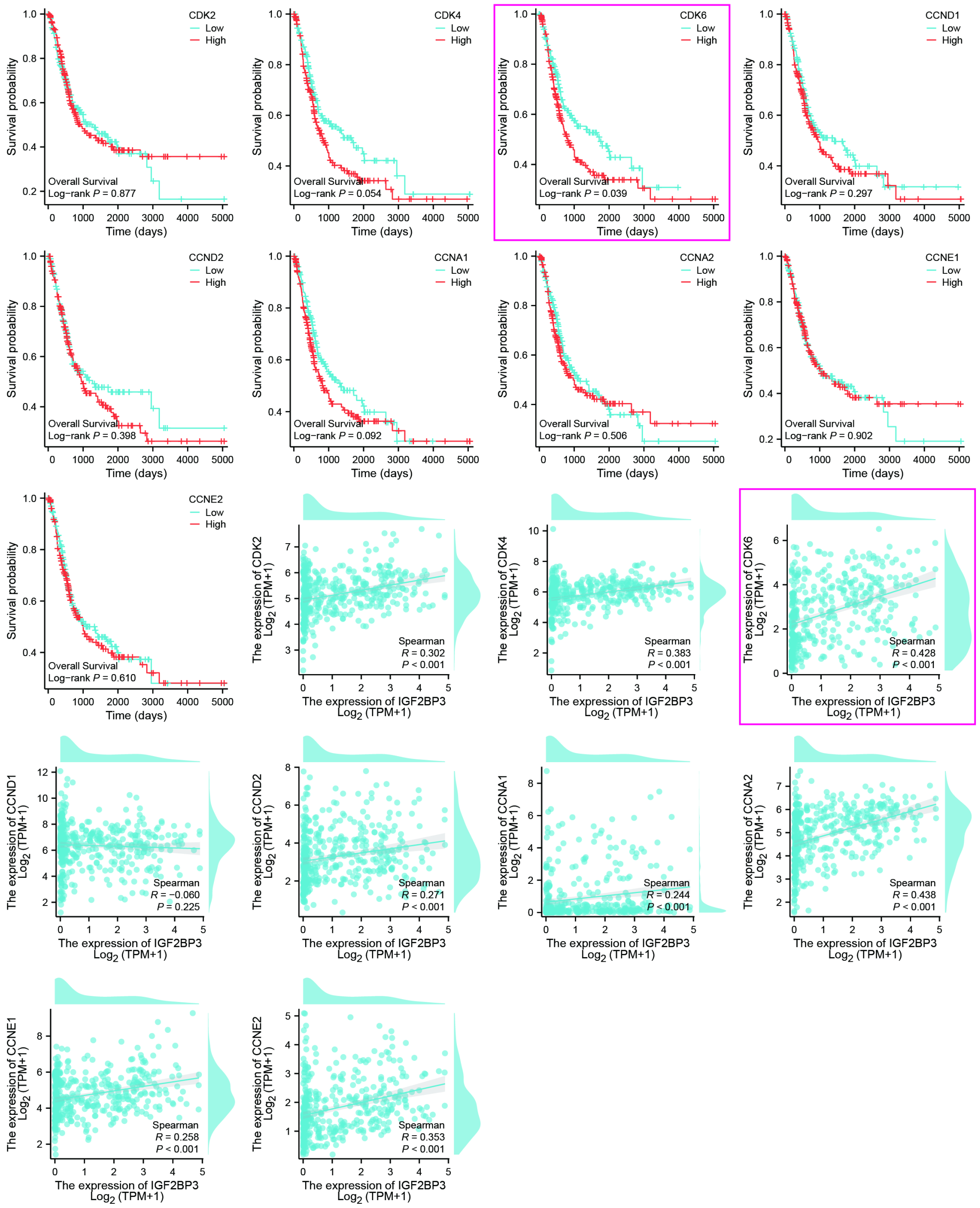

FigureS3: Survival analysis and co-expression analysis to validate the association between key regulators of the cell cycle pathway and IGF2BP3 from TCGA dataset.

(A) A significant correlation between overexpression of CDK6 and poor survival prognosis for patients with bladder cancer (P=0.039). (B)A strong relationship between expression levels of CDK6 and IGF2BP3 (R = 0.428, *P* < 0.001).

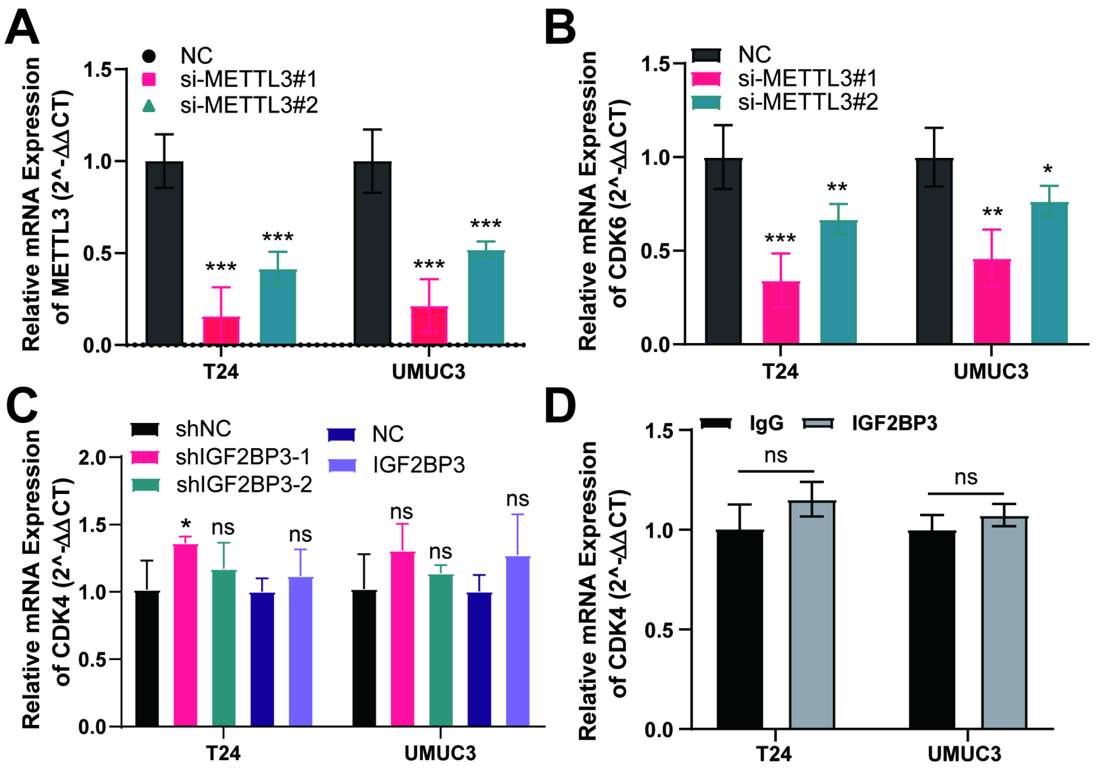

FigureS4: METTL3 targeted CDK6 mRNA.

(A)METTL3 was knockdown in bladder cancer cell lines (****P*< 0.001). (B) A positive correlation between METTL3 and CDK6 expression was observed in METTL3 knockdown bladder cancer cell lines (**P*< 0.05). (C) Non-significant correlation between IGF2BP3 and CDK4 expression was observed in bladder cancer cell lines (**P*< 0.05; ns:non-significant). (D) RIP assay demonstrated that anti-IGF2BP3 antibodies didn't enriched CDK4 mRNA compared to IgG antibodies (ns:non-significant)

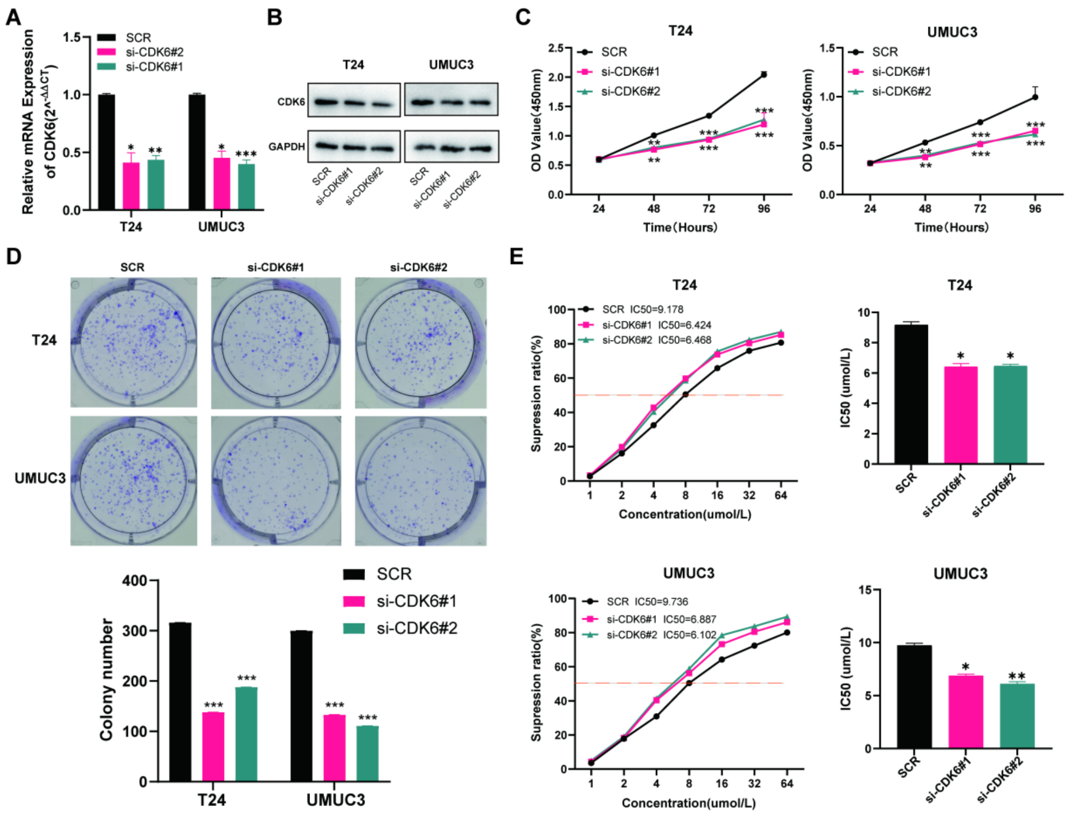

FigureS5: CDK6 interference decreased the cell proliferation and increased cisplatin chemotherapy sensitivity in bladder cancer cells.

(A-B) The transfection efficiency of CDK6 interference was verified by qRT- PCR (A) and western blot assays (B) (**P*<0.05, ***P*<0.01, ****P*< 0.001). (C) CCK-8 assay results showed that knocking down CDK6 resulted in a significant decrease in T24 and UMUC3 growth rate (***P*<0.01, ****P*< 0.001). (D) The colony-formation assays result also confirmed interference CDK6 decreased the colony formation in T24 and UMUC3 cells (****P*< 0.001). (E) Knockdown of CDK6 can increase cisplatin chemotherapy sensitivity in bladder cancer cells. (**P*<0.05, ***P*<0.01)

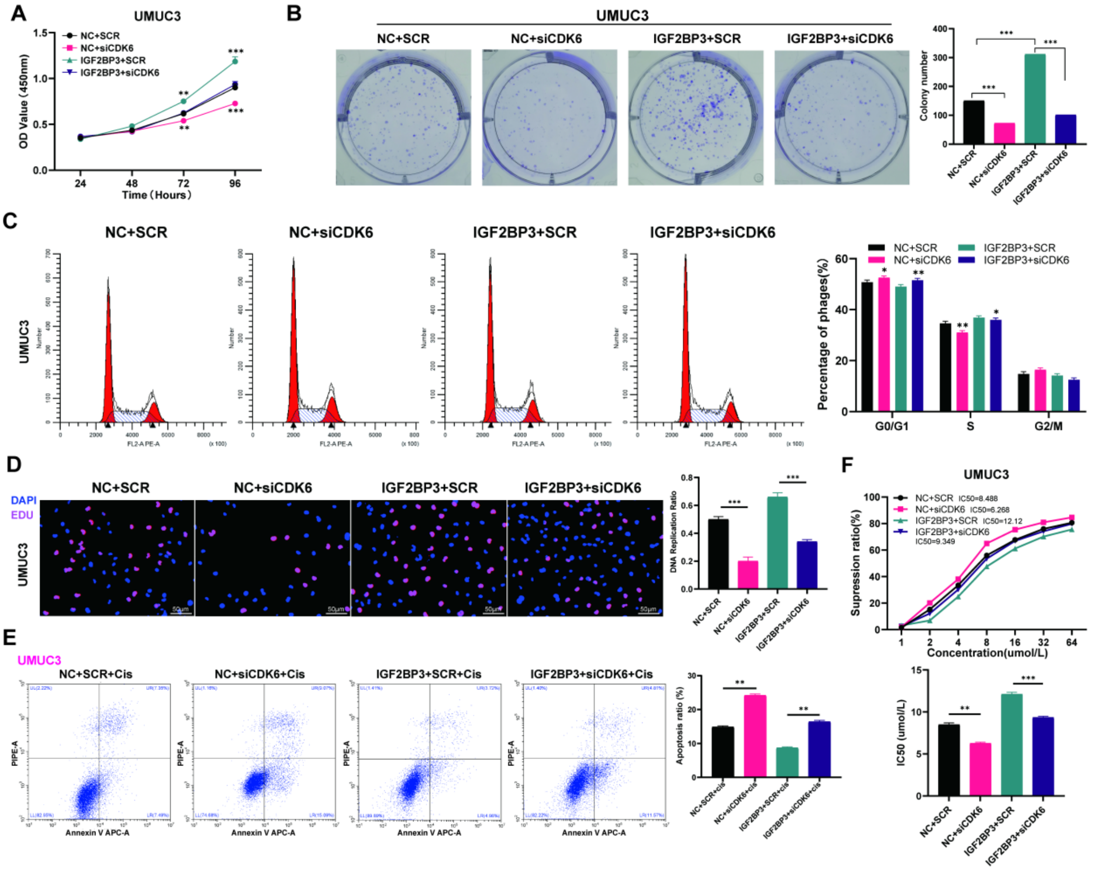

FigureS6: CDK6 interference decreased the cell proliferation and increased the extent of apoptosis induced by IGF2BP3 in UMUC3 cells.

(A) Knockdown of CDK6 reversed IGF2BP3 induced proliferation in UMUC3 cells (***P*<0.01, ****P*< 0.001). (B) Cloning experiments produced consistent results to CCK-8 assay (****P*< 0.001). (C) Knocking down CDK6 reversed G1 phase arrest induced by overexpression of IGF2BP3 (**P*<0.05, ***P*<0.01). (D) EDU assay demonstrated that knockdown of CDK6 expression reversed the DNA replication capacity of bladder cancer cells induced by IGF2BP3 (****P*< 0.001). (E) Knockdown of CDK6 could significantly increase the apoptosis rate of bladder cancer cells induced by IGF2BP3 (***P*<0.01). (F) Knocking down CDK6 expression could significantly reduce the IC50 of cisplatin in UMUC3 that were elevated due to over-expression of IGF2BP3 (***P*<0.01, ****P*< 0.001).

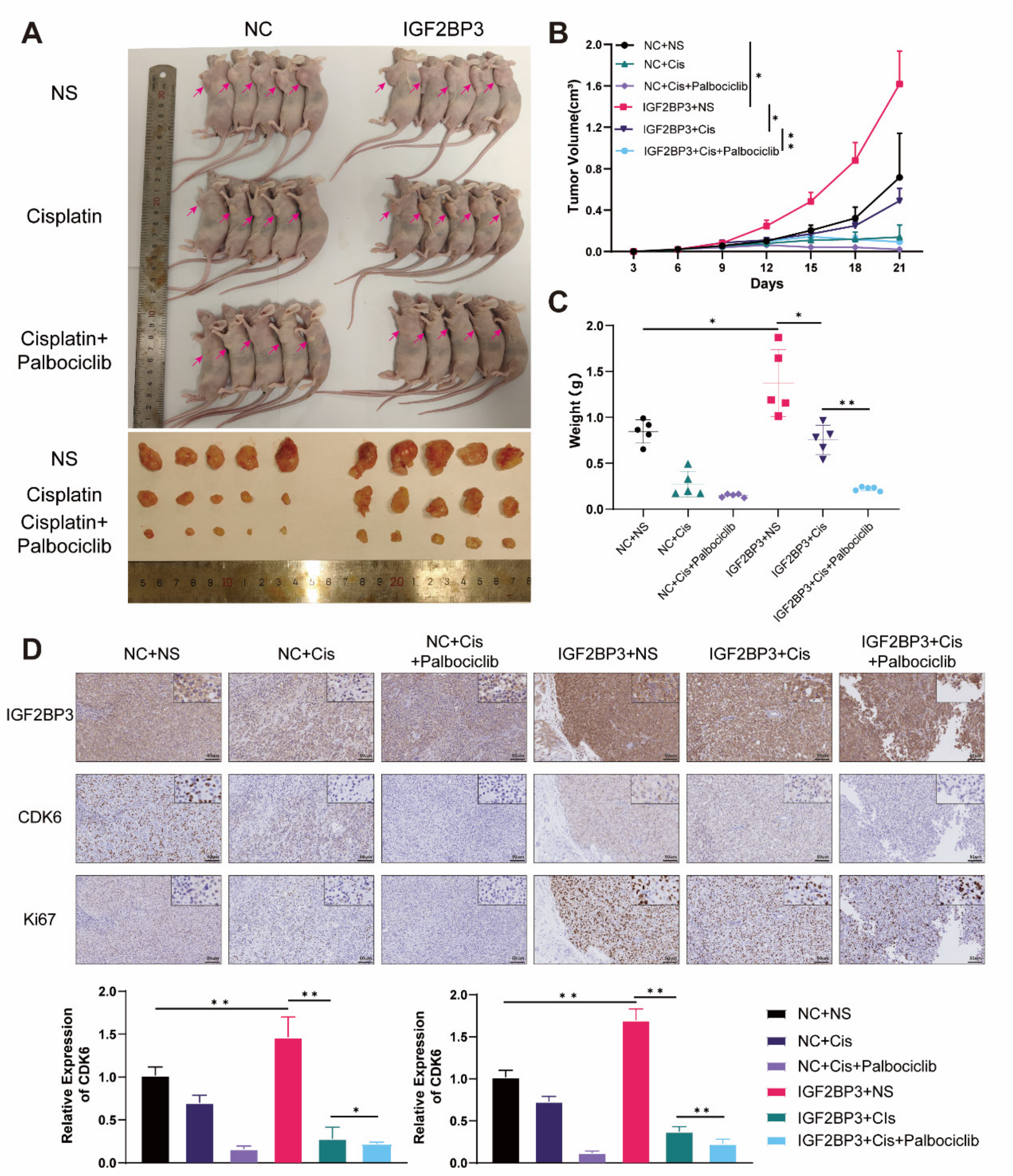

FigureS7: Palbociclib enhances sensitivity to cisplatin chemotherapy in *vivo.*

(A) The nude mice with IGF2BP3 overexpression (IGF2BP3) and control cells (NC) were intraperitoneally injected with cisplatin or normal saline, or co-injected with cisplatin and Palbociclib, respectively, on the 7th day after implantation. The red arrow indicates the tumor location. (B-C) The tumor volume (B) and weight (C) were significantly increased in the IGF2BP3 overexpression group (IGF2BP3) compared with the normal saline control group (NC). The sensitivity of cisplatin chemotherapy was inhibited by IGF2BP3. Palbociclib could improve sensitivity to cisplatin chemotherapy. (D) Immunohistochemical analysis of CDK6 and Ki67 expression in

tumors from different groups (*$P<0.05$, **$P<0.01$, ns: non-significant; Cis:cisplatin).
