# Supplementary material for: IGF2BP3 promotes the proliferation and cisplatin resistance of bladder cancer by enhancing the mRNA stability of CDK6 in an m6A dependent manner
Source: Int J Biol Sci. 2025 Feb 18;21(5):2048–66. doi: 10.7150/ijbs.103522 (PMC11900814; doi:10.7150/ijbs.103522)
Supplement: Supplementary file 3 — Supplementary table 2. [file ijbsv21p2048s3.pdf]

Table S2. Primers used in this research (5'-3')

Primes	Sequences
β-Actin forward	CAAATTCCATGGCACCGTC
β-Actin reverse	TCTCGCTCCTGGAAGATGGT
GAPDH forward	CAGGAGGCATTGCTGATGAT
GAPDH reverse	GAAGGCTGGGGCTCATTT
IGF2BP3 forward	ACTGCACGGGAAACCCATAG
IGF2BP3 reverse	ACTATCCAGCACCTCCCACT
CDK6 forward	GCTGACCAGCAGTACGAATG
CDK6 reverse	GCACACATCAAACAACCTGACC
CDK4- forward	ATGGCTACCTCTCGATATGAGC
CDK4- reverse	CATTGGGGACTCTCACACTCT
METTL3 forward	AAGCTGCACTTCAGACGAAT
METTL3 reverse	GGAATCACCTCCGACACTC
CDK6-MeRIP forward	AGAGGCCGACTGACACTCGCA
CDK6-MeRIP reverse	ACACGATTACATAGCCTATGCC
